# Effect of the aggregated protein dye YAT2150 on *Leishmania* parasite viability

**DOI:** 10.1128/aac.01127-23

**Published:** 2024-02-13

**Authors:** Lucía Román-Álamo, Yunuen Avalos-Padilla, Inés Bouzón-Arnáiz, Valentín Iglesias, Jorge Fernández-Lajo, Juan M. Monteiro, Luis Rivas, Roser Fisa, Cristina Riera, David Andreu, Carlos Pintado-Grima, Salvador Ventura, Elsa M. Arce, Diego Muñoz-Torrero, Xavier Fernàndez-Busquets

**Affiliations:** 1 Barcelona Institute for Global Health (ISGlobal), Hospital Clínic-Universitat de Barcelona, Barcelona, Spain; 2 Institute for Bioengineering of Catalonia (IBEC), The Barcelona Institute of Science and Technology, Barcelona, Spain; 3 Doctoral School of Biotechnology, Faculty of Pharmacy and Food Sciences, University of Barcelona, Barcelona, Spain; 4 Institut de Biotecnologia i de Biomedicina (IBB) and Departament de Bioquímica i Biologia Molecular, Universitat Autònoma de Barcelona, Bellaterra, Spain; 5 Centro de Investigaciones Biológicas Margarita Salas, Consejo Superior de Investigaciones Científicas (CSIC), Madrid, Spain; 6 Section of Parasitology Department of Biology, Health and Environment, Faculty of Pharmacy and Food Sciences, University of Barcelona, Barcelona, Spain; 7 Department of Medicine and Life Sciences, Barcelona Biomedical Research Park, Pompeu Fabra University, Barcelona, Spain; 8 Laboratory of Medicinal Chemistry (CSIC Associated Unit), Faculty of Pharmacy and Food Sciences, and Institute of Biomedicine (IBUB), University of Barcelona, Barcelona, Spain; 9 Nanoscience and Nanotechnology Institute (IN2UB), University of Barcelona, Barcelona, Spain; The Children's Hospital of Philadelphia, Philadelphia, Pennsylvania, USA

**Keywords:** antileishmanial drugs, *Leishmania*, protein aggregation, YAT2150

## Abstract

The problems associated with the drugs currently used to treat leishmaniasis, including resistance, toxicity, and the high cost of some formulations, call for the urgent identification of new therapeutic agents with novel modes of action. The aggregated protein dye YAT2150 has been found to be a potent antileishmanial compound, with a half-maximal inhibitory concentration (IC_50_) of approximately 0.5 µM against promastigote and amastigote stages of *Leishmania infantum*. The encapsulation in liposomes of YAT2150 significantly improved its *in vitro* IC_50_ to 0.37 and 0.19 µM in promastigotes and amastigotes, respectively, and increased the half-maximal cytotoxic concentration in human umbilical vein endothelial cells to >50 µM. YAT2150 became strongly fluorescent when binding intracellular protein deposits in *Leishmania* cells. This fluorescence pattern aligns with the proposed mode of action of this drug in the malaria parasite *Plasmodium falciparum*, the inhibition of protein aggregation. In *Leishmania major*, YAT2150 rapidly reduced ATP levels, suggesting an alternative antileishmanial mechanism. To the best of our knowledge, this first-in-class compound is the only one described so far having significant activity against both *Plasmodium* and *Leishmania*, thus being a potential drug for the treatment of co-infections of both parasites.

## INTRODUCTION

Leishmaniasis is a neglected tropical disease caused by protozoan parasites of the *Leishmania* genus, of which more than 20 species can infect humans and other mammals (1). The parasite is transmitted to people through the bite of phlebotomine sandflies, where it is found as extracellular promastigotes, which are eventually phagocytized by macrophages, where they transform into the intracellular amastigote form. Depending on the infecting species and the host immune response, three main clinical manifestations of the disease arise: cutaneous leishmaniasis (CL), mucocutaneous leishmaniasis, and visceral leishmaniasis (VL). Visceral clinical manifestation is the most severe form of the disease and has a ca. 95% mortality rate if untreated. Estimates indicate a global annual incidence of 0.9 to 1.6 million cases of CL and around 90,000 of VL, most of them affecting low-income countries (2). In the European and African Mediterranean regions, *Leishmania infantum* and *Leishmania major* are, respectively, the main species causing a pathological condition. Current treatments for leishmaniasis vary depending on the clinical form but face several challenges, including drug resistance, toxicity concerns, administration as painful injections into the lesions, parenteral delivery, and the high cost of certain formulations (3). The lack of optimal treatment options has prompted the World Health Organization to prioritize the search for new therapeutic targets and drugs as critical for leishmaniasis eradication efforts (4).

Protein misfolding and aggregation are regarded as the molecular bases for numerous human pathologies, ranging from neurodegenerative diseases to cancer and type II diabetes (5). In these cases, toxicity arises from either the absence of properly functioning polypeptides or the formation of cytotoxic oligomers and/or fibrils. On the bright side, protein aggregation serves functional purposes in various organisms, e.g., as a rapid mechanism to respond to environmental challenges through stress granule formation (6) or uncovering genetic diversity needed in starvation (7), to facilitate the controlled release of peptidic hormones in secretory granules in a pH-dependent manner (8), or to promote synapse-specific changes that strengthen dendritic spines and contribute to memory consolidation (9). Recently, we described that in the human malaria parasite *Plasmodium falciparum*, protein aggregation presumably plays a functional role and that its inhibition is detrimental to the pathogen (10, 11). Motivated by these findings, we have explored in the current work the therapeutic potential of inhibiting protein aggregation for other parasitic diseases. We evaluated the effect of the aggregated protein dye YAT2150, a potent antiplasmodial drug with a half-maximal inhibitory concentration (IC_50_) of ca. 90 nM in *in vitro P. falciparum* cultures (11), on the viability of *L. infantum* promastigotes and amastigotes. Malaria and leishmaniasis are the first and second parasitic diseases, respectively, in number of deaths (12), in many cases with overlapping distribution, although the clinical study of co-infection with both pathogens has been mostly neglected (13). A good antileishmanial activity of YAT2150 could postulate it as a drug of choice for the treatment of malaria and leishmaniasis co-infections.

## RESULTS AND DISCUSSION

### Detection of protein aggregation in live *L. infantum* cells

**Fig 1:**
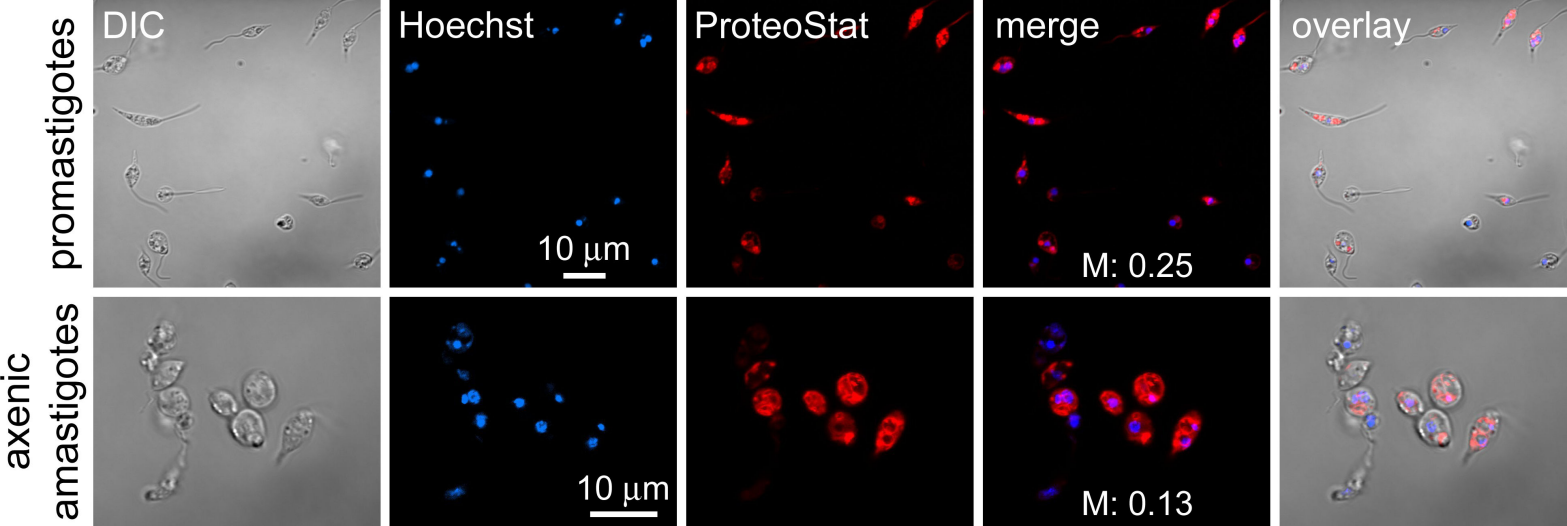
Detection of intracellular protein aggregates in live *L. infantum* promastigotes and axenic amastigotes by confocal fluorescence microscopy using ProteoStat. M, Manders’ correlation coefficients indicating the fraction of ProteoStat co-localized with Hoechst 33342; DIC, differential interference contrast image.

Previous studies demonstrated the effectiveness of the red fluorescent dye ProteoStat to detect intracellular protein aggregates in live cells of the malaria parasite *P. falciparum* (10). However, a straightforward extrapolation of such analysis into *Leishmania* faces stark contrast to the theoretical predictions made for these two protozoa. Aggregation-prone proteomes usually result from genomes with a high proportion of AT bases, which in *P. falciparum* is 80.6% (14), while approximately 37.5% in *L. major* and *L. infantum* (15). Moreover, low-complexity regions, known to promote protein aggregation, constitute 12.2% of the entire genome in the *Leishmania donovani* complex (*L. donovani* and *L. infantum*) (16), while in *P. falciparum*, they represent *ca*. 49% of it (17). Finally, the predicted content in the *L. infantum* proteome of prion-like proteins (directly correlated with aggregation propensity) is merely 0.95% according to the PLAAC algorithm (18), significantly lower than for *P. falciparum* (9.40%) (19). Despite these differences, live *L. infantum* promastigotes and axenic amastigotes were strongly stained with ProteoStat (Fig. 1). The Manders’ correlation coefficient with the DNA dye Hoechst 33342 indicated a preferentially non-nuclear localization of the ProteoStat signal. These findings from *in vitro* cultures evidenced protein aggregation as a prevalent phenomenon in *L. infantum*.

### Identification of aggregation-prone proteins in *L. infantum* promastigotes

**Fig 2:**
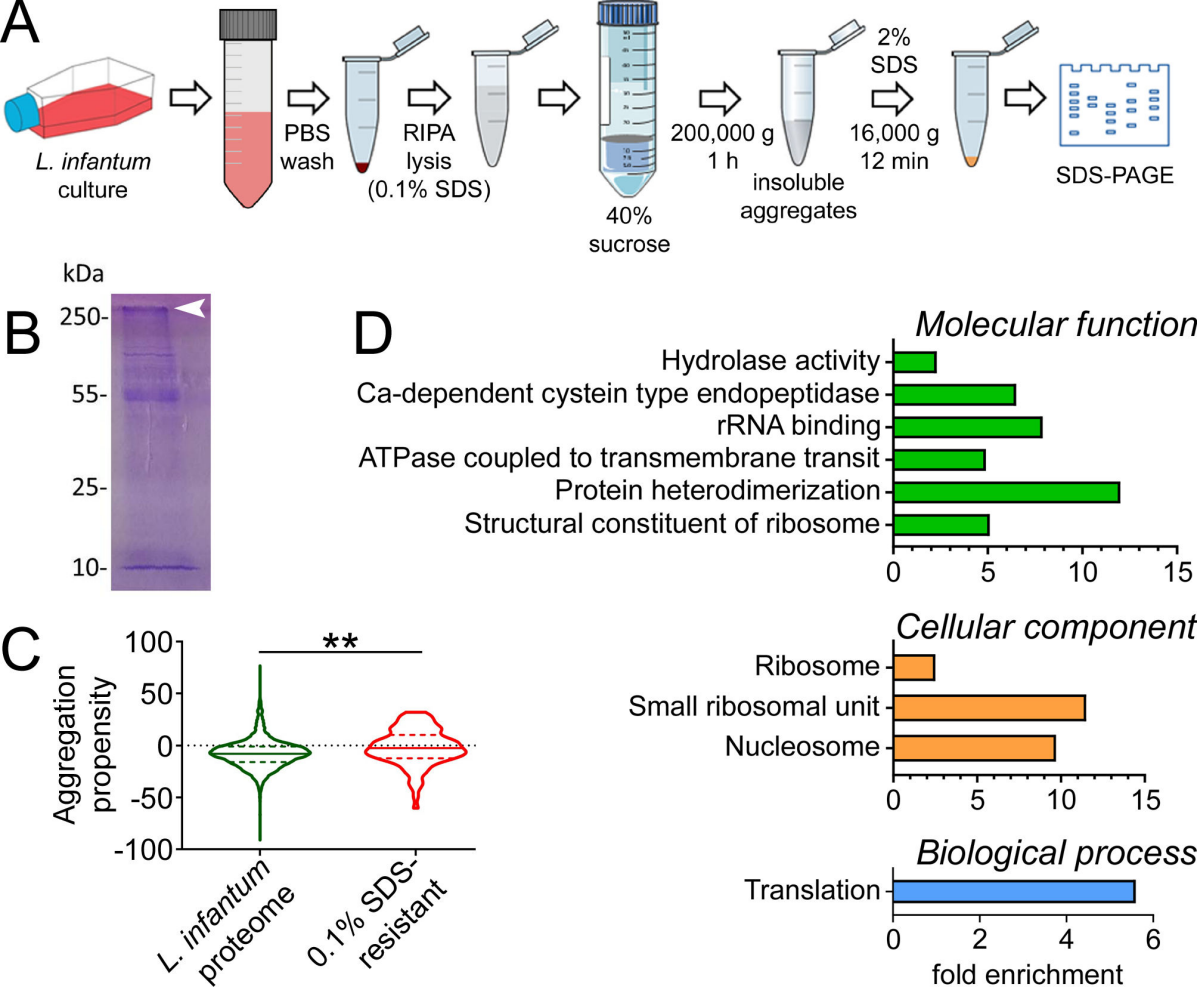
Isolation of *L. infantum* proteins insoluble in 0.1% SDS. (**A**) Scheme of the process. (**B**) Coomassie Blue-stained SDS-PAGE fractionation of the 0.1% SDS-resistant sample. The arrowhead indicates the excised region subjected to LC-MS/MS analysis. (**C**) Aggregation propensity [Aggrescan score (21)] of the *L. infantum* proteins resisting solubilization in 0.1% SDS (excluding membrane proteins) vs. the proteome of the parasite. **P < 0.005. (**D**) GO enrichment analysis of the 92 non-membrane *L. infantum* proteins identified in 0.1% SDS-resistant aggregates, classified according to biological process, cellular component, and molecular function.

To confirm that the observed ProteoStat staining reflected the presence of intracellular protein aggregates, we proceeded to identify aggregation-prone proteins present in live parasites. With that aim, a homogenate from *L. infantum* promastigotes resisting dissolution in the presence of 0.1% sodium dodecyl sulfate (SDS) was loaded in a polyacrylamide gel electrophoresis (PAGE), and the Coomassie Blue-stained material not entering the separating gel was excised and subjected to liquid chromatography with tandem mass spectrometry analysis (LC-MS/MS) (Fig. 2A and B). A total of 132 *L*. *infantum* proteins were identified (Table S1), which included a number of membrane proteins that in live cells are protected from aggregation as their hydrophobic stretches are mostly found inside a lipid bilayer. Therefore, proteins annotated as membrane or transmembrane in the UniProt database (20) were removed (Table S1, shadowed in gray), obtaining a group of 92 proteins (Table S1, non-shadowed), whose propensity to aggregate, as expected, was significantly higher than that of the rest of the *Leishmania* proteome (Fig. 2C).

To functionally characterize the non-membrane proteins found in 0.1% SDS-resistant aggregates, a Gene Ontology (GO) enrichment analysis was performed (22). The enriched categories (Fig. 2D and Table S2) were mainly related to translation, with the ribosome, small ribosomal subunit and nucleosome as main cellular components, and protein heterodimerization activity, rRNA binding, and structural constituent of ribosome as some of the most represented molecular functions. Ribosomal activity is of singular importance in *Leishmania* since the absence of a regulated transcription by RNA polymerase II leaves the control of gene expression at the translational level (23). Ribosomal proteins often have a high cytoplasmic abundance in concentrations above their solubility limit, existing in a metastable state protected from aggregation by kinetic barriers and assisted by chaperones (24). It is conceivable that changing conditions such as the concentration of molecular components or the disruption of ribosomal complex assembly provoked by internal or external stresses, like changes in temperature, pH, or the presence of drugs, can affect the aggregation state of ribosomal proteins, thereby influencing parasite viability. Several studies have demonstrated a lack of correlation between protein abundance and mRNA levels in certain *Leishmania* species, as regulation predominantly occurs through post-transcriptional and post-translational mechanisms (25). Given this context, it is tempting to speculate that the parasite modulates the abundance of its ribosomal components, to either facilitate or impede their aggregation according to specific physiological requirements.

### *In vitro* characterization of the aggregation of peptides selected from the live *L. infantum* aggregation-prone protein pool

Protein aggregation relies heavily on substantial sequential homology, and even minor mutations influence aggregation reactions (26). As the initial amyloid nucleation step can be expedited when preformed fibrils of the same protein or shorter variants containing the amyloid driver are present (27), highly amyloidogenic peptides found in *L. infantum* proteins with high aggregation propensity may exacerbate protein aggregation in the pathogen. To identify these peptides, proteins resistant to dissolution in 0.1% SDS were analyzed by machine-learning techniques for modeling protein tertiary structure (28) to discern peptides exposed to the solvent in their native state. Two peptides were selected, DNFIFGQ from the β chain of tubulin, a major microtubule component, and AISVFFLEP from the cleavage and polyadenylation specificity factor (CPSF)-like protein, involved in mRNA processing. Notably, both sequences exhibited high amyloidogenic propensity based on the amyloid prediction method WALTZ (29) (Table 1) and solvent exposure according to the AlphaFold structural model (30) (Fig. S1).

**TABLE 1:** Amyloidogenic peptides selected from the aggregation-prone *L. infantum* protein pool non-solubilized by 0.1% SDS

Protein (UniProt accession)	Peptide sequence	WALTZ score
Tubulin β chain (A0A381MS01)	_88_DNFIFGQ_94_	91.97
CPSF (A4I7V5)	_663_AISVFFLEP_671_	94.46

**Fig 3:**
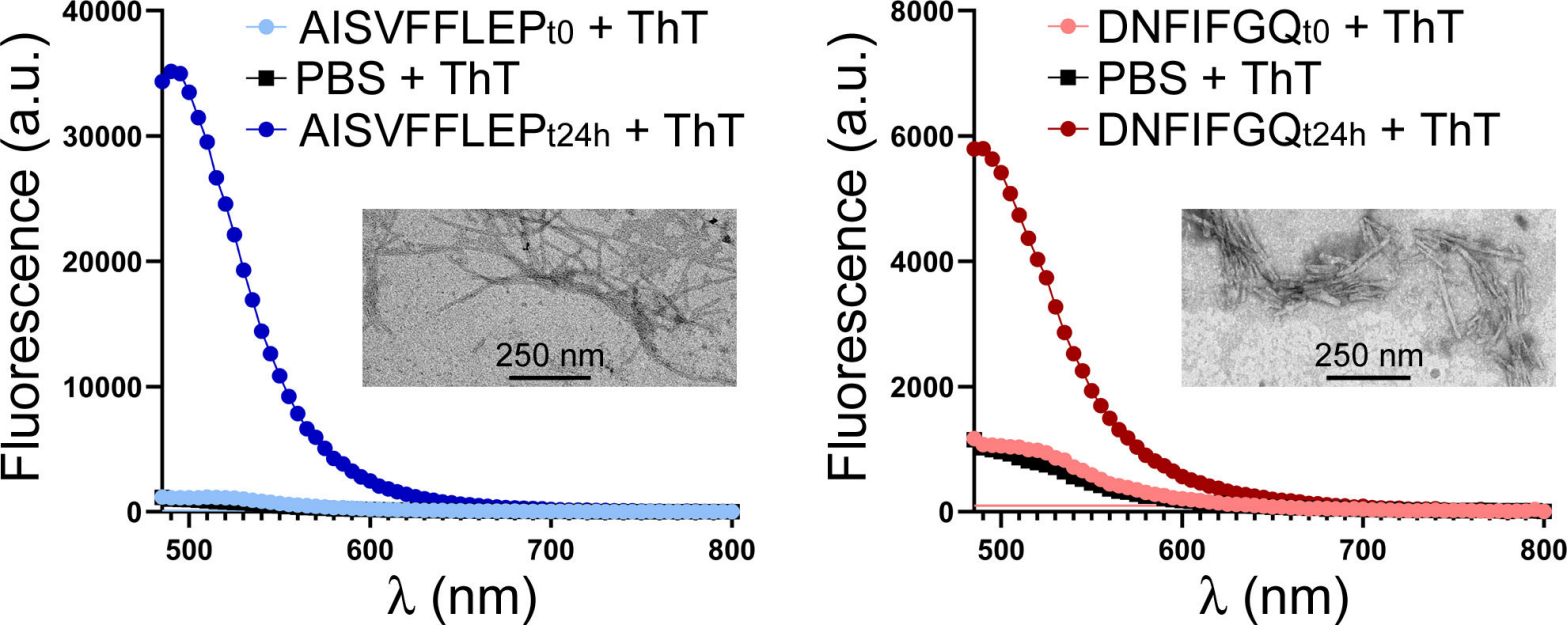
Characterization of the *in vitro* aggregation of peptides AISVFFLEP and DNFIFGQ. Representative TEM images (insets) and ThT fluorescence analyses are shown for 25 µM and 180 µM concentrations of both peptides, respectively. a.u., arbitrary units.

The *in vitro* aggregation of peptides DNFIFGQ and AISVFFLEP was confirmed by transmission electron microscopy (TEM) imaging and analysis of thioflavin T (ThT) fluorescence emission, an indicator of amyloid-like aggregation (31) (Fig. 3). Stretches of five or more continuous amino acids from the sequences of both peptides, especially for AISVFFLEP, are found in other *L. infantum* proteins (Table S3), which could contribute to boost their potential aggregative properties in live cells. Nevertheless, treatment for 72 h of *L. infantum* promastigotes with up to 100 µM of either peptide did not significantly affect the viability of this form of the parasite (2% and 0% growth inhibition in the presence of 100 µM DNFIFGQ and AISVFFLEP, respectively), despite both peptides bound and entered promastigotes according to flow cytometry and confocal fluorescence microscopy analysis (Fig. S2). Previous studies have succeeded with targeted aggregation in live plant cells by inducing the endogenous expression of aggregation-prone peptides, obtaining a visible specific phenotype (27). However, similar attempts failed to induce toxicity in *P. falciparum* (11) or human cell lines (32), likely by the difficulty of attaining a critical concentration of seeds necessary to sustain intracellular aggregation of the desired protein, discouraging this approach as a therapeutic alternative in *Leishmania*.

### Effect of protein aggregation inhibitors in *L. infantum* cultures

**Fig 4:**
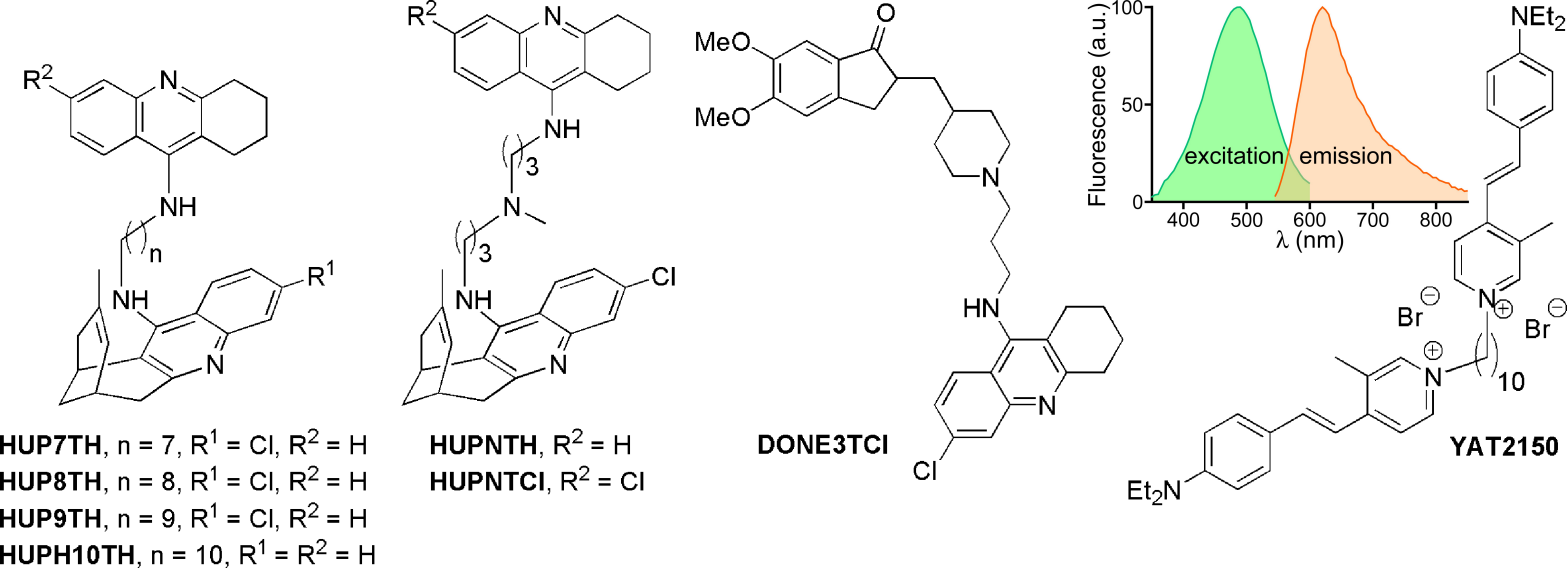
Chemical structures of different compounds which interfere with protein aggregation (33) and inhibit the growth of *P. falciparum* (11). The inset shows the fluorescence excitation/emission spectrum of YAT2150.

The detection in live *L. infantum* of protein aggregates and the identification in the parasite of proteins containing amyloidogenic peptides mirrored similar phenomena recently observed in *P. falciparum* (10). In the latter pathogen, low concentrations (<100 nM) of certain β-sheet intercalators, which lowered the *in vitro* aggregation of amyloid peptides (33), clearly inhibited its growth (11). When testing the effect on *L. infantum* of some of these small molecules known to interfere with protein aggregation (Fig. 4), they significantly reduced the parasite’s viability (Table 2; Fig. S3). One of these compounds, YAT2150, stood out with an *in vitro* IC_50_ of around 0.52 µM against both promastigotes and amastigotes, which was ca. 54-, 20-, and 542-fold lower than that reported in promastigotes for the commonly used antileishmanial agents pentamidine (34), miltefosine (35), and paromomycin (36), respectively. Amphotericin B has a lower IC_50_ (0.04 µM in promastigotes) (37), but frequently associated nephrotoxicity has limited its clinical use, a drawback absent in its liposomal formulations (38). The half-maximal cytotoxic concentration (CC_50_) of YAT2150 for human umbilical vein endothelial cells was 3.4 ± 0.5 µM, which resulted in a modest selectivity index (SI: CC_50_/IC_50_) of approximately 7.

**TABLE 2:** Effect of the compounds from Fig. 4 on the viability of *L. infantum* promastigotes and amastigotes

	IC_50_ promastigotes (µM)	IC_50_ amastigotes (µM)	CC_50_^*a*^ (µM)	Selectivity index^*b*^ (CC_50_/IC_50_)
YAT2150	0.52 ± 0.07	0.51 ± 0.11	3.4 ± 0.5	6.6
DONE3TCl	2.09 ± 0.40	0.62 ± 0.08^*c*^	12.6 ± 2.2	6.0
HUPNTCl	2.03 ± 0.19	1.04 ± 0.21^*c*^	6.3 ± 0.5	3.1
HUPNTH	2.37 ± 1.40	1.52 ± 0.66^*c*^	3.4 ± 1.0	1.4
HUP7TH	1.23 ± 0.42	0.92 ± 0.38^*c*^	7.8 ± 3.4	6.4
HUP8TH	0.99 ± 0.47	1.63 ± 0.34^*c*^	4.9 ± 1.3	4.9
HUP9TH	0.88 ± 0.29	0.57 ± 0.13^*c*^	4.9 ± 1.0	5.6
HUPH10TH	0.72 ± 0.11	0.97 ± 0.21^*c*^	3.4 ± 0.1	4.7

^*a*^ Cytotoxicity assayed in human umbilical vein endothelial cells.

^*b*^ For promastigotes.

^*c*^ *n* = 2.

**Fig 5:**
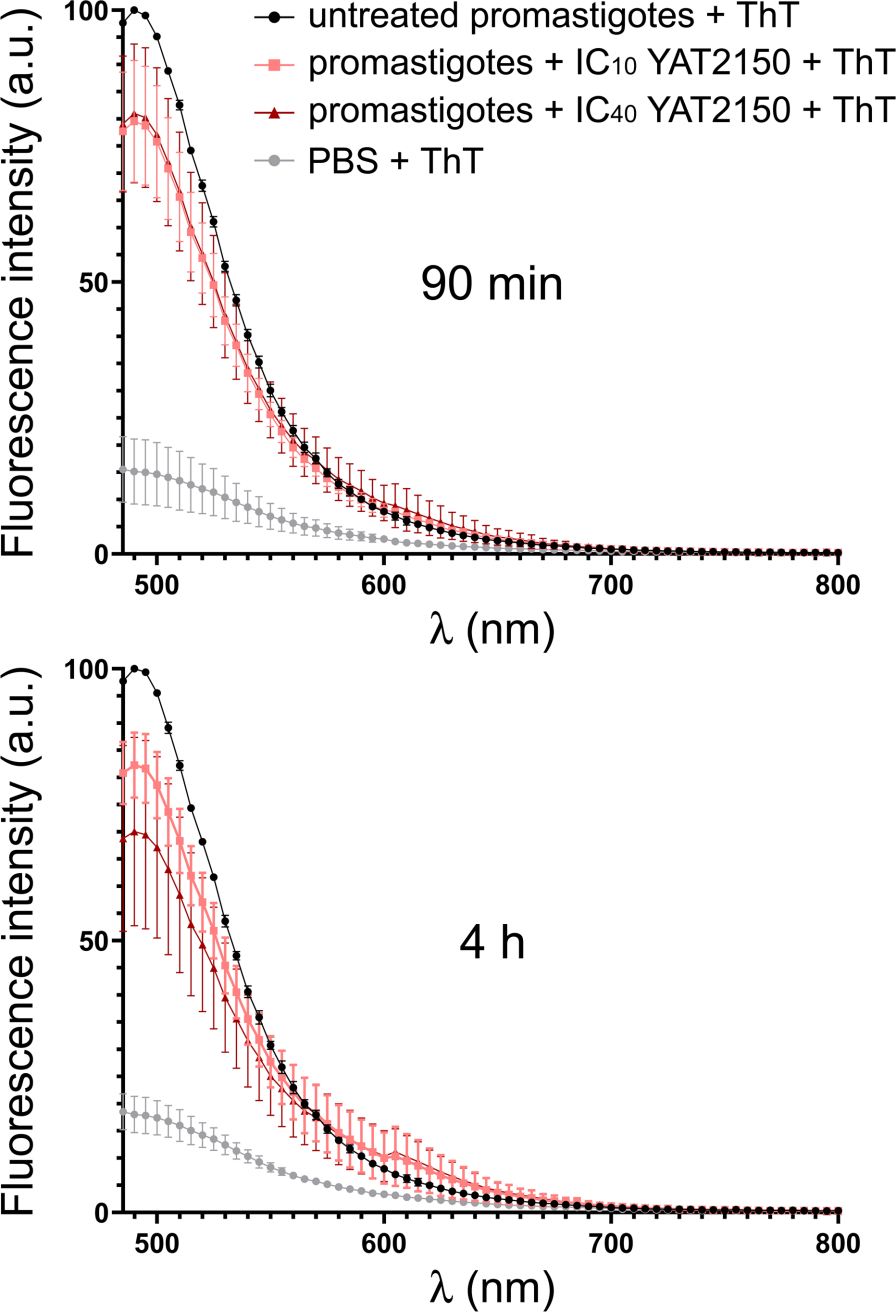
Thioflavin T (ThT) fluorescence of *L. infantum* culture extracts adjusted to have equal protein content, either non-treated or treated with YAT2150 at its *in vitro* IC_10_ (0.15 µM) and IC_40_ (0.3 µM), for 90 min and 4 h (*n* = 3). The arbitrary units (a.u.) of fluorescence intensity are normalized relative to a value of 100 assigned to the untreated promastigotes + ThT sample. Error bars indicate standard deviation.

*Leishmania* differentiation from promastigotes to amastigotes is induced by acid pH and high temperature (39, 40), two factors which increase protein aggregation (41, 42). The antileishmanial effect of certain peptide aggregation inhibitors on amastigotes might be related to the upsetting of some essential differentiation mechanism in the parasite. In agreement with its proposed activity as a protein aggregation inhibitor in *P. falciparum* (11), at physiologically relevant concentrations below its *in vitro* IC_50_, YAT2150 reduced in *L. infantum* promastigote cultures the overall aggregation of the parasite’s proteins according to ThT analysis (Fig. 5). This result suggested that one of the antileishmanial mechanisms of action of this compound might be related to interfering with the aggregative state of certain proteins in the pathogen. The possibility that such interference hampers particular protein-protein interactions, which might be part of, e.g., essential transcription factor networks whose disruption would compromise parasite viability, remains to be explored in detail.

### Physical and pharmacological properties and early safety profiling of YAT2150

To explore the potential of YAT2150 to become a therapeutic agent, its druglikeness has been addressed through a preliminary assessment of physical and drug metabolism and pharmacokinetics (DMPK) properties and of its tolerability (Table 3). The solubility of YAT2150, measured at 37°C in phosphate-buffered saline (PBS) containing 1% dimethyl sulfoxide (DMSO), was found to be 8.5 µM, which indicated a favorable aqueous solubility at a concentration of up to 16-fold higher than its IC_50_. YAT2150 is moderately lipophilic, with a calculated logP below 5 (clogP = 3.37), i.e., compliant with Lipinski’s rule of five (43). Some pharmacokinetic parameters related to intestinal absorption and metabolism were also determined. The intestinal absorption of YAT2150 was assessed using the Caco-2 permeability assay, commonly used to predict the *in vivo* absorption of drugs. The transport across a Caco-2 cell monolayer was determined in both directions, so that not only absorption but also active efflux could be determined. These assays demonstrated a high absorption (>200 nm/s) and moderate efflux (ca. 100 nm/s) for YAT2150, resulting in a favorable efflux ratio of 0.48. The metabolic stability of YAT2150 was determined in human plasma, human microsomes, and human hepatocytes. When incubated at 37°C with human plasma from healthy donors, YAT2150 was perfectly stable after 2 h and 91% of the compound still remained unchanged after 6 h of incubation. YAT2150 showed also a good stability in human microsomes: 55% of the compound remained unchanged after 1 h of incubation at 37°C, displaying a half-life of 80 min and a microsomal intrinsic clearance (CLint) of 10.6 µL/min/mg protein, i.e., below 15 µL/min/mg protein, thereby behaving as a low clearance compound (44). The stability of YAT2150 in human hepatocytes was still higher, with 70% of it remaining unchanged after 2 h of incubation at 37°C, a longer half-life of 277 min and a low intrinsic clearance (2.5 µL/min/10^6^ cells). Overall, YAT2150 has quite favorable metabolic stability and should not be eliminated too fast from the organism. With regard to the safety profile, apart from its moderate selectivity index relative to human umbilical vein endothelial cells, the inhibitory effects of YAT2150 on some cytochrome P450 (CYP) isoforms commonly involved in drug metabolism were evaluated. At 10 µM, i.e., a 20-fold higher concentration than its IC_50_ against *L. infantum* promastigotes, YAT2150 inhibited CYP1A2 and CYP2C9 by around 50%, while the inhibition of CYP2C19 and CYP2D6 was two- and threefold less potent, respectively, than its antileishmanial effect. Despite the overall good DMPK profile, the eventual future pharmacological development of YAT2150 will probably require its optimization through the synthesis of improved derivatives (45), in a similar way as it has been reported for, e.g., DNDI-6148, a preclinical candidate for the treatment of VL (46).

**TABLE 3:** DMPK and early safety properties of YAT2150

Physicochemical properties			
Molecular mass	832.85 Da		
Aqueous solubility^*a*^	8.5 µM		
Lipophilicity (clogP)^*b*^	3.37		

Pharmacokinetic parameters			
Intestinal absorption			
	Papp AB (nm/s)	Papp BA (nm/s)	Efflux ratio
Caco-2 permeability^*c*^	207.6 ± 20.1	100.6 ± 0.4	0.48
Metabolic stability			
	% remaining^*d*^	*t*_1/2_ (min)^*e*^	CLint^*f*^
Plasma stability^*g*^	100.0% after 1 h 99.7% after 2 h 91.3% after 6 h		
Microsomal stability^*g*^	54.6% after 1 h	80.5	10.6 µL/min/mg protein
Hepatocyte stability^*g*^	70.4% after 2 h	277.3	2.5 µL/min/10^6^ cells

Safety profiling (CYP inhibition)^*h*^			
CYP1A2	55% ± 1% inhibition at 10 µM		
CYP2C9	51% ± 4% inhibition at 10 µM		
CYP2C19	IC_50_ = 1.1 µM		
CYP2D6	IC_50_ = 1.7 µM		

^*a*^ Kinetic solubility at 37°C in PBS containing 1% DMSO.

^*b*^ Calculated using Molinspiration.

^*c*^ Transport of compound from A→B (AB) and B→A (BA) in Caco-2 cells incubated at 37°C; apparent permeability (Papp) values in nm/s; efflux ratio = Papp BA/Papp AB.

^*d*^ Percentage unchanged compound after a given incubation time.

^*e*^ Half-life in min.

^*f*^ Intrinsic clearance.

^*g*^ Stability of the compound in human plasma, human microsomes, or human hepatocytes at 37°C.

^*h*^ Inhibition potential of YAT2150 using human recombinant cytochrome P450 enzymes at 37°C.

### Effect of YAT2150 on *L. major* ATP levels

Due to the experimental availability of promastigotes of a strain of *L. major* (a species associated with cutaneous leishmaniasis) used as a real-time *in vivo* sensor for variation of intracellular ATP levels (47), we tested energy metabolism as a potential target for YAT2150. The extrapolation of the results of this approach to *L. infantum* relies on the relative promiscuity of the interactions underlying the protein aggregation phenomenon and the high homology between *L. infantum* and *L. major* proteomes. A comparative UniProt BLAST analysis between both species of the proteins in Table S1 showed amino acid sequence homology exceeding 90% for most of them, with only 7 out of 132 proteins below 80%.

**Fig 6:**
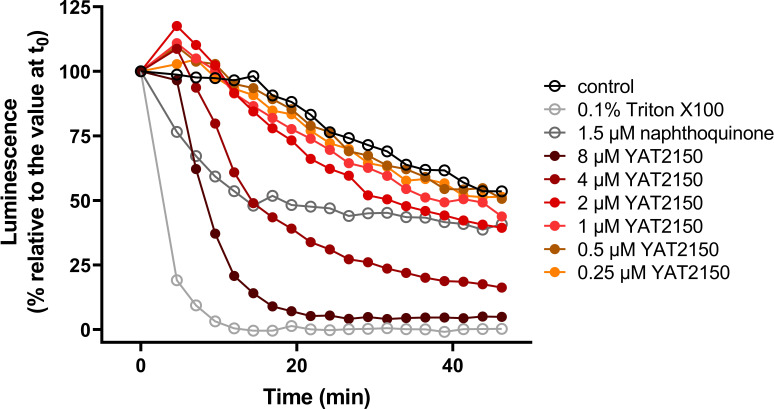
Luciferase assay for the assessment of free intracellular ATP levels in YAT2150-treated *L. major* promastigotes.

For the determination of ATP levels, promastigotes expressing a cytoplasmic form of firefly luciferase were used, and, to overcome the poor membrane permeability of luciferin, the membrane-permeable derivative 1-(4,5-dimethoxy-2-nitrophenyl)ethyl ester (DMNPE)-luciferin was employed. Under these conditions, ATP is the limiting substrate for the luminescence reaction in living parasites. Upon exposure to YAT2150, a concentration-dependent reduction in luminescence was observed for concentrations beyond 2 µM (Fig. 6). The disparity between this threshold concentration for YAT2150 and its IC_50_ may likely obey to the higher density of promastigotes required in the ATP experiment, as, presumably, YAT2150 operates via a stoichiometric mechanism to disrupt or prevent the formation of aggregated structures. Furthermore, a rapid response was sought to ensure that the decline in ATP levels represented a primary target rather than a consequence of overall microorganism deterioration. Currently, it remains unclear whether the decrease of protein aggregation and the drop in ATP are interconnected or independent of each other. In the former hypothesis, it could be postulated that physiological aggregation serves as a protective mechanism against a potentially toxic protein fragment or peptide, which could disrupt the energy metabolism of *Leishmania*. In the latter scenario, the existence of an additional target for YAT2150 might complicate the rational design of new analogs, but, on the other side, it would also hamper the evolution of resistance against this molecule.

### Fluorescence microscopy analysis of YAT2150 staining of *L. infantum*

**Fig 7:**
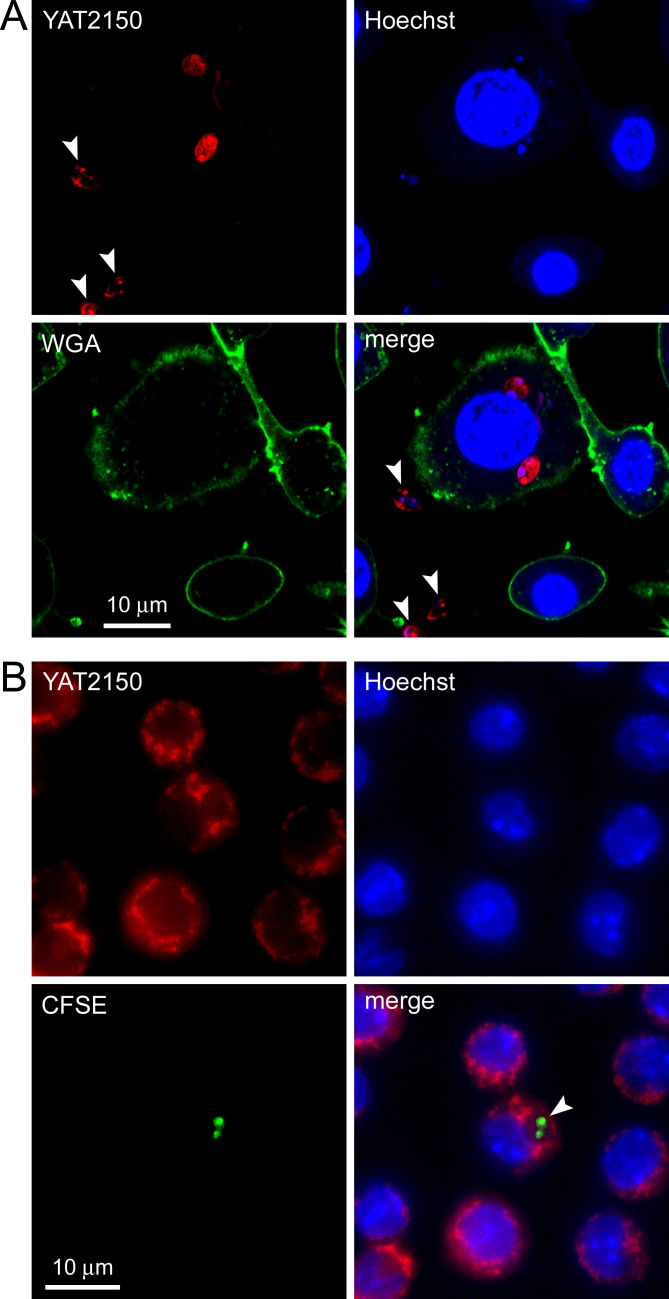
Fluorescence microscopy analysis of YAT2150 staining of *L. infantum* promastigote-exposed RAW 264.7 macrophages. (**A**) Confocal fluorescence microscopy images of live macrophages incubated for 24 h in the presence of *L. infantum* promastigotes that had been pre-stained for 1 h with 0.38 µM YAT2150. Cell membranes were stained with Oregon Green-conjugated wheat germ agglutinin (WGA). Arrowheads indicate promastigotes outside macrophages. (**B**) Fluorescence microscopy image of live macrophages exposed to CFSE-stained *L. infantum*. Nuclei were stained for 10 min with 2 µg/mL Hoechst 33342 and protein aggregates for 6 h with 0.38 µM YAT2150. The arrowhead indicates a *Leishmania*-containing phagolysosome.

As expected from being the main component of ProteoStat, the fluorescence of YAT2150 was strong in *L. infantum* promastigotes and axenic amastigotes (Fig. S4). YAT2150-stained promastigotes were observed in macrophages that had been incubated in their presence for 24 h (Fig. 7A). However, it could not be unequivocally determined whether these intracellular parasites were amastigotes, recently phagocytosed promastigotes, or intermediate forms. When macrophages exposed to *L. infantum* promastigotes that had been stained with carboxyfluorescein succinimidyl ester (CFSE) were treated for 6 h with YAT2150, sufficient to stain vesicular structures in the cytosol of macrophages, no YAT2150 signal could be observed in the phagolysosomes containing *Leishmania* (Fig. 7B). Although this result might indicate a potential lack of permeability of YAT2150 across phagolysosome membranes, it could also be due to a loss or significant reduction of intracellular protein aggregates in *Leishmania* parasites in phagolysosomes, in a similar way as it has been described in *P. falciparum* following its invasion of red blood cells (10, 11). The presence in *L. infantum* promastigotes of an aggregated protein coat might be a protective barrier for the parasite in the hostile extracellular environment, not required anymore inside the phagolysosome. Another possible function of an aggregated protein structure in promastigotes could be related to interactions with certain macrophage receptors required as part of the phagocytosis mechanism. Overall, YAT2150 might be staining dynamic protein associations that are required in promastigotes but not in intracellular amastigotes, where they would progressively disassemble. In agreement with this hypothesis, it has been established that the surface coat of amastigotes is notably less complex than that of promastigotes (48).

### *In vitro* activity on *L. infantum* growth of YAT2150 encapsulated in liposomes

**Fig 8:**
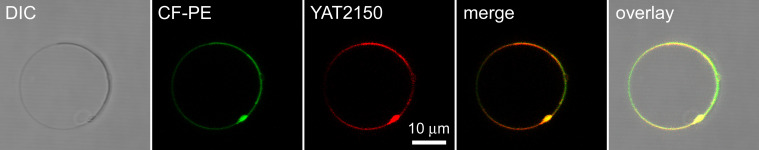
Confocal fluorescence microscopy analysis of the encapsulation of 2.5 µM YAT2150 in GUVs (10 mM total lipid concentration; see Materials and Methods for GUV lipid composition). To stain the lipid bilayer, the lipid carboxyfluorescein-phosphatidylethanolamine (CF-PE) was included in the GUV formulation. DIC, differential interference contrast image.

In addition to the synthesis of chemical derivatives mentioned above, a second strategy commonly used to improve the selectivity index of drugs is their encapsulation in targeted nanocarriers. Here, to increase the therapeutic window of YAT2150, its incorporation into liposomes was assayed. Targeted delivery offers the potential of reducing drug cytotoxicity through encapsulation (which screens non-specific interactions) and increasing antiparasitic potency through the local accumulation in or near *Leishmania* cells. Building upon our previous success in enhancing the activity of antimalarials through encapsulation in targeted immunoliposomes (49), a similar approach for YAT2150 was taken. This strategy benefits from the lipophilic properties of YAT2150 to undergo a preferential partition into the lipid bilayer of liposomes, as shown using giant unilamellar vesicles (GUVs) as a membrane model (Fig. 8).

Drug-encapsulating liposomes were prepared with an estimated final concentration of 60 µM YAT2150 (for 10 mM total lipid), corresponding to an encapsulation efficiency of 60%. YAT2150-loaded liposomes showed a growth inhibition activity against *L. infantum* amastigotes and promastigotes higher than that of the free drug (Table 4). As an attempt to increase specificity toward *Leishmania* cells, an antibody raised against lipophosphoglycan (LPG), the major parasite surface glycoconjugate which is present in large numbers in promastigotes and to a lesser extent in amastigote-containing macrophages (50) (Fig. S5), was conjugated to liposomes (Fig. S6). However, the incorporation of anti-LPG antibodies to liposomes did not improve drug activity in amastigotes and did it only marginally in promastigotes, suggesting that LPG might not be an adequate antigen for targeted drug delivery approaches against *Leishmania*. The unspecific cytotoxicity of liposome-encapsulated YAT2150 decreased to a CC_50_ > 50 µM, thus increasing the SI of this drug formulation to above 100. Encapsulation of YAT2150 might offer the additional benefit of minimizing its interaction with cell membranes, thus contributing to reduce its presumably high biodistribution, which would be an important issue regarding an eventual future pharmaceutical development.

**TABLE 4:** Determination of diameter, polydispersity index, IC_50_ in *L. infantum* promastigotes and amastigotes, and CC_50_ of YAT2150 encapsulated in liposomes and in immunoliposomes functionalized with antibodies against LPG

	Diameter (nm)	Polydispersity index	IC_50_ promastigotes (µM)	IC_50_ amastigotes (µM)	CC_50_ (µM)	SI
YAT2150 in solution	‒	‒	0.52 ± 0.07	0.51 ± 0.11	3.4 ± 0.5	6.6
YAT2150-liposomes	154.7 ± 1.8	0.113 ± 0.025	0.37 ± 0.01^*a*^	0.19 ± 0.02^*b*^	>50	>100
YAT2150-immunoliposomes	155.3 ± 0.9	0.130 ± 0.009	0.34 ± 0.02^*b*^	0.35 ± 0.08	>50	>100

^*a*^ *P* < 0.05.

^*b*^ *P* < 0.005.

**Fig 9:**
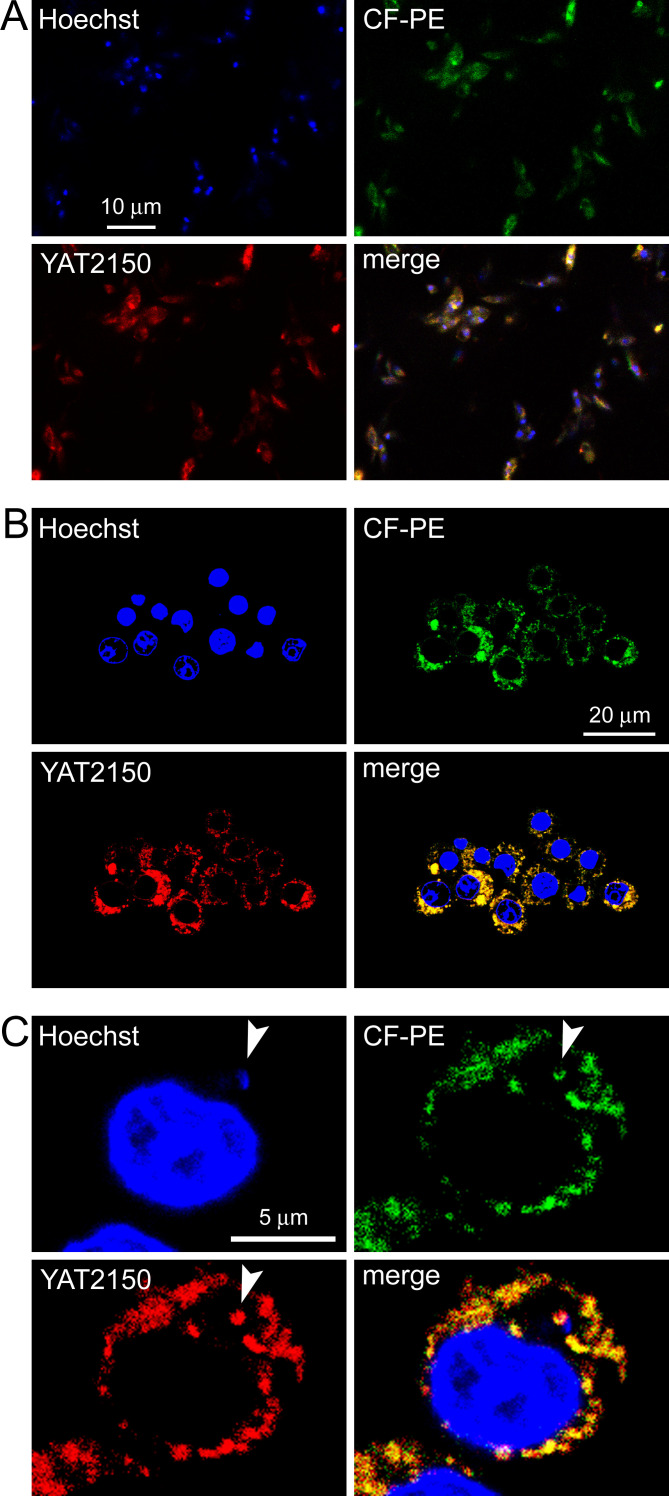
Confocal fluorescence microscopy analysis of the cell targeting of 0.38 µM YAT2150 encapsulated in fluorescein-labeled anti-LPG immunoliposomes. (**A**) Live *L. infantum* promastigotes were incubated for 1 h with immunoliposomes, stained for 20 min with 4 µg/mL Hoechst 33342 to reveal nuclei, and fixed with 3% paraformaldehyde. (**B, C**) RAW 264.7 macrophages that had been exposed to *L. infantum* promastigotes were incubated for 3 h with immunoliposomes, stained for 10 min with 2 µg/mL Hoechst 33342, and fixed. The green fluorescence corresponds to CF-PE incorporated in the liposome formulation. The arrowhead indicates a *Leishmania* amastigote inside its phagolysosome.

As expected, confocal fluorescence microscopy analysis of live *L. infantum* cells treated with YAT2150 encapsulated in liposomes (Fig. S7) and anti-LPG immunoliposomes (Fig. 9) showed the drug’s fluorescence associated to promastigotes (Fig. 9A) and in parasite-treated macrophages (Fig. 9B). However, in this case, YAT2150 fluorescence could be occasionally observed co-localizing with the parasite inside phagolysosomes (Fig. 9C), perhaps reflecting an improved entry facilitated by liposome encapsulation.

### Conclusions

Treatment of *L. infantum* cultures with peptide aggregation inhibitors showed a significant *in vitro* antileishmanial effect. In particular, the compound YAT2150 had a better *in vitro* activity against *L. infantum* (IC_50_ of ca. 0.5 µM for both amastigote and promastigote forms) than some drugs currently used for the clinical treatment of leishmaniasis, such as miltefosine, pentamidine, and paromomycin. Encapsulation in liposomes of YAT2150 significantly improved the *in vitro* antileishmanial activity relative to the free drug and reduced its unspecific cytotoxicity, which led to a SI > 100 and therefore to good perspectives regarding preclinical assays of this compound or of its eventual future derivatives having less toxicity and/or higher antiparasitic activity. The antileishmanial mode of action of YAT2150 might be multipronged, with protein aggregation and ATP biosynthesis as potential cellular targets. The properties of YAT2150 that postulate it as an excellent drug candidate against *Plasmodium* and *Leishmania* hold promise for its use in the treatment of co-infections of both pathogens.

## MATERIALS AND METHODS

### Reagents

Except where otherwise indicated, reagents were purchased from Sigma-Aldrich Corporation (St. Louis, MO, US), and reactions were performed at room temperature (22°C–24°C). The tested compounds were synthesized as previously described (11, 51–53).

### Data acquisition and bioinformatics analysis

The reference *L. infantum* proteome (JPCM5, proteome ID UP000008153) was downloaded from UniProt [release 2022_01 (20)]. The subcellular location of *L. infantum* proteins was extracted from UniProt annotations and manually cured. Transmembrane annotated proteins were analyzed with DeepTMHMM v1.0.15, an improved version of TMHMM 2.0 [https://dtu.biolib.com/DeepTMHMM/ (54)], capable of identifying transmembrane beta-barrels. Propensity to protein aggregation was assessed using the Aggrescan prediction method (21), and the Normalized a4vSS for 100 residues (Na4vSS) was acquired as the aggregation reporter. Sequences over 6000 residues were divided into smaller fragments, and the Na4vSS for the whole ensemble was calculated. For the *L. infantum* full proteome, the mean aggregation propensity is displayed. The content in prion-like proteins was assessed with the PLAAC algorithm (18) using the whole proteome for each case as the background probability, and those proteins with at least one window with COREscore > 0 were considered prion-like. Structures for *L. infantum* tubulin β chain (A0A381MS01) and cleavage and polyadenylation specificity factor-like protein (A4I7V5) were obtained from the AlphaFold database (30) version 3 and colored with Pymol. Amyloid prediction was carried out with Waltz (29) in its default “best overall performance” mode. GO enrichment analysis was performed with the functional annotation tool of the Database for Annotation, Visualization and Integrated Discovery knowledge base update v2022q3 (22), where GO terms annotated directly by the source (GO_DIRECT category) were selected and the *L. infantum* JPCM5 data set was chosen as background.

### Peptide synthesis

Acetyl-DNFIFGQ-amide and acetyl-AISVFFLEP-amide were synthesized at a 0.1-mmol scale on H-Rink amide-ChemMatrix resin (PCAS BioMatrix Inc., Saint-Jean-sur-Richelieu, QC, Canada) in a Prelude (Gyros Protein Technologies AB, Uppsala, Sweden) synthesizer running Fmoc solid-phase peptide synthesis (SPPS) protocols. After chain assembly, the N-terminus was deprotected and acetylated (acetic anhydride/*N*,*N*-diisopropylethylamine, 1 mmol each, 45 min, dimethylformamide), prior to full deprotection and cleavage with trifluoroacetic acid (TFA)/H_2_O/triisopropyl-silane (95:2.5:2.5 vol/vol), 90 min, at room temperature. Peptides were precipitated from the TFA solution by the addition of cold diethyl ether followed by centrifugation (3 × 4800 rpm, 5 min, 4°C), taken up in water, and lyophilized. Crude peptides were checked by analytical reverse-phase high-performance liquid chromatography (RP-HPLC) and liquid chromatography-mass spectrometry (LC-MS) and purified by preparative RP-HPLC. Analytical RP-HPLC was performed on a LC-20AD instrument (Shimadzu Corporation, Kyoto, Japan) equipped with a Luna C18 column (3 µm, 4.6 mm × 50 mm; Phenomenex, Torrance, CA, US) using linear gradients of solvent B [0.036% TFA in acetonitrile (ACN)] into A (0.045% TFA in H_2_O) over 15 min, at a flow rate of 1 mL/min and UV detection at 220 nm. Preparative RP-HPLC was performed on a LC-8 instrument (Shimadzu Corporation) fitted with a Luna C18 column (10 µm, 21.2 mm × 250 mm; Phenomenex), using linear gradients of solvent D (0.1% TFA in ACN) into C (0.1% TFA in H_2_O) over 30 min, with a flow rate of 25 mL/min. MS analysis was performed on a LC-MS 2010EV instrument (Shimadzu Corporation) fitted with an XBridge C18 column (3.5 µm, 4.6 mm × 150 mm; Waters, Cerdanyola del Vallès, Spain), eluting with linear gradients of F [0.08% formic acid (FA) in ACN] into E (0.1% FA in H_2_O) over 15 min at a 1-mL/min flow rate. Fractions with the expected mass and >95% purity by LC-MS were pooled and lyophilized. Peptide stock solutions were prepared in sterile deionized water and stored at ‒20°C. Fluorescent versions of the peptides were prepared by coupling 5(6)-carboxyfluorescein to the N-terminus of the peptide resins, as described for acetylation. The fluorescein(Flu)-labeled Flu-DNFIFGQ-amide and Flu-AISVFFLEP-amide were obtained after resin cleavage, preparative RP-HPLC purification, and LC-MS characterization as above.

### *In vitro* peptide aggregation assays

To completely disaggregate the peptides before the assay, around 15 mg of each lyophilized peptide was dissolved in 1 mL of TFA. After thoroughly mixing, TFA was evaporated under a N_2_ stream and 0.5 mL of 1,1,1,3,3,3-hexafluoro-2-propanol (HFIP) was added, mixed well, and evaporated as before (repeated twice to fully remove TFA). Then, 2 mL of HFIP was added, 40-nM peptide aliquots were prepared in low-binding Eppendorf tubes (Eppendorf, Hamburg, Germany), and HFIP was evaporated overnight in a desiccator. For aggregation assays, peptide stocks were prepared in PBS at a final concentration of 200 µM peptide and incubated at 37°C under stirring at 1,400 rpm in a ThermoMixer (Eppendorf) for 24 h (*t*_24h_). Then, in a 96-well flat-bottom black plate (Greiner Bio-One, Frickenhausen, Germany), the peptide solutions were diluted in PBS and ThT was added to obtain final concentrations of 180 µM peptide and 25 µM ThT. The fluorescence emission of ThT was collected from 460 to 800 nm using an excitation wavelength of 450 nm (Infinite Nano+ multimode microplate reader; Tecan Trading AG, Männedorf, Switzerland). As a non-aggregation control, the ThT fluorescence of a freshly disaggregated 180 µM peptide solution (*t*_0_) was measured.

### Transmission electron microscopy

Peptides (25 µM) were incubated in PBS at 37°C for 24 h under orbital stirring at 300 rpm. After 5 min of water bath sonication (FB15053 ultrasonic bath; Thermo Fisher Scientific Inc., Waltham, MA, US), a 5-µL drop of the peptide solution was deposited onto a carbon-coated copper grid (Ted Pella, Redding, CA, US). Next, 5 µL of 2% uranyl acetate was added and left for 5 min before washing it away with H_2_O. The resulting samples were observed with a JEM 1010 transmission electron microscope (JEOL Ltd., Tokyo, Japan). Images were acquired using an Orius 832 CCD camera (Gatan Inc., Pleasanton, CA, US).

### Encapsulation of YAT2150 in liposomes

Liposomes were prepared by the thin lipid film hydration method (55). Briefly, the lipids 1,2-dioleoyl-*sn*-glycero-3-phosphocholine (DOPC):1,2-distearoyl-*sn*-glycero-3-phosphoethanolamine-*N*-[maleimide(polyethylene glycol)−2000] (DSPE-PEG-Mal):cholesterol (75:5:20 molar ratio) were mixed in chloroform:methanol (2:1 vol/vol) in a glass vial together with 100 µM YAT2150 from a YAT2150 stock solution in DMSO. Organic solvents were removed by evaporation under nitrogen. The thin lipid film formed on the vial walls was hydrated in PBS, in a volume corresponding to a final lipid concentration of 10 mM. Then, three rounds of constant vortexing for 2 min followed by bath sonication at 35°C for 3 min (FB15053 ultrasonic bath) were performed. Liposomes were extruded through 200 nm polycarbonate membranes (Avanti Polar Lipids Inc., Alabaster, AL, US) using a mini extruder device (Avanti Polar Lipids Inc.). Non-encapsulated YAT2150 was removed by pelleting liposomes by ultracentrifugation (150,000 × *g*, 1 h, 4°C) and replacing the supernatant by PBS, except in the case of cytotoxicity assays, where the pellet was taken up in Medium 199 (M-199) supplemented with 1% penicillin-streptomycin. Fluorescein-labeled liposomes containing a molar ratio of 0.5% CF-PE were prepared as described above, except for a reduced DOPC content of 74.5%. The average diameter and polydispersity index of liposomes were measured after 1:100 sample dilution in PBS, using a Zetasizer NanoZS90 (Malvern Ltd., Malvern, UK). To determine the drug content, liposomes were disrupted by treatment with one volume of 4% SDS, and YAT2150 was quantified by absorbance spectroscopy at a wavelength of 490 nm (Epoch microplate spectrophotometer; BioTek Instruments Inc., Winooski, VT, US). Encapsulation efficiencies are defined as the fraction of YAT2150 in the liposome pool, after ultracentrifugation, relative to the initial amount of drug added to the liposomes. The amount of YAT2150 in the sample is expressed relative to the total lipid concentration of 10 mM, assuming that all the lipids end up in liposomes.

### Immunoliposome preparation and characterization

Immunoliposomes were prepared by attaching the anti-lipophosphoglycan (LPG) monoclonal IgM antibody CA7AE (Bio-Rad, Hercules, CA, US) to the maleimide group of DSPE-PEG-Mal following established protocols (49). The *N*-succinimidyl *S*-acetylthioacetate crosslinker (SATA; Thermo Fisher Scientific Inc.) was first coupled to the antibody at a molar ratio of 1:10 (1 mg antibody/mL in 350 µL of PBS) through an incubation step of 30 min at room temperature. Non-reacted SATA was removed by buffer exchange. Thioester groups from SATA-anti-LPG antibody were deacetylated with 0.1 volumes of deacetylation solution (0.5 M hydroxylamine, 25 mM EDTA in PBS) and incubated for 2 h at room temperature. Then, another buffer exchange was performed with PBS containing 10 mM EDTA to remove the remaining deacetylation solution. To 100 µL (10 mM total lipid in PBS) of a suspension of liposomes exposing maleimide groups and carrying YAT2150, were added 55 µL of thiolated SATA-anti-LPG antibody and 45 µL of PBS supplemented with 10 mM EDTA. The resulting sample was incubated for 17 h at room temperature, when unbound SATA-anti-LPG antibody and YAT2150 were removed by ultracentrifugation (150,000 × *g*, 1 h, 4°C) and replaced with PBS containing 10 mM EDTA. The YAT2150 concentration in immunoliposomes was quantified as specified above.

The presence of anti-LPG antibody and YAT2150 in liposomes was assessed by SDS-PAGE. Ten microliters of each sample was mixed with 10 µL of 2× Laemmli sample buffer (0.125 M tris base, 4% SDS, 20% glycerol, 10% 2-mercaptoethanol, and 2 mg/mL bromophenol blue) and heated at 95°C for 5 min. Then, the samples were run in a 12% SDS-PAGE (Mini Protean II System; Bio-Rad), and the gel was stained with silver nitrate following the next steps: fixation (40% ethanol, 10% acetic acid) for 30 min, washing three times with deionized water (Milli-Q system; Millipore Corporation, Burlington, MA, US), staining for 40 min (1 mg/mL silver nitrate, 0.02% formaldehyde), and developing (25 mg/mL Na_2_CO_3_, 0.01% formaldehyde) until visualization of the protein bands, when 1% acetic acid was used to stop the reaction. Then, the YAT2150 fluorescent signal was detected in a LAS 4000 reader (ImageQuant TL; GE Healthcare, Chicago, IL, US) with the Cy3 filter, and an image of the protein bands was taken by white epi-digitalization.

### Gel-assisted formation of giant unilamellar vesicles

GUVs were produced by the gel-assisted method with minor modifications (56). A 5% (wt/wt) polyvinyl alcohol (PVA, 145 kDa; Merck, Darmstadt, Germany) solution was prepared in PBS at 90°C and used to coat a microscope coverslip which was later dried for 30 min in an oven at 50°C. Then, 20 µL of the lipid formulation (10 mM total lipid) DOPC:cholesterol:CF-PE (79.5:20:0.5 molar ratio) was spread over the dried PVA. To evaporate the solvent, the glass slide was placed under vacuum for 30 min at room temperature. Two coverslips and a Teflon spacer were assembled forming a chamber (2-mL volume) that was filled with PBS. One hour later, GUVs were harvested and YAT2150 was added at a final concentration of 2.5 µM. Fifteen microliters of the resulting GUV suspension was transferred to a 2% bovine serum albumin (BSA)-coated coverslip and observed with a Leica TCS SP5 laser scanning confocal fluorescence microscope (Leica Microsystems, Wetzlar, Germany). Fluorescein and YAT2150 were detected by excitation at 488 and 561 nm and emission collection in ranges 497–543 and 572–657 nm, respectively.

### Cytotoxicity assays

Human umbilical vein endothelial cells were maintained in M-199 supplemented with 10% fetal bovine serum (FBS) and 1% penicillin-streptomycin at 37°C, 5% CO_2_, and seeded in 96-well plates at a density of 50,000 cells/mL. After allowing cell adherence for 24 h, the medium was removed, and drugs or nanoformulations were added in M-199 supplemented with 1% penicillin-streptomycin. Plates were incubated for 48 h, and then, the medium in each well was replaced by 100 µL of M-199 containing penicillin-streptomycin and 0.00125% resazurin sodium salt (Sigma-Aldrich Corporation). Plates were incubated for 4 h, and resorufin fluorescence emission was measured (λex/em: 535/590 nm) in a Tecan Infinite 200 PRO equipment (Tecan Trading AG).

### Solubility screen assay

The stock solutions (10^‒2^ M) of the assayed compounds were diluted to decreased molarity, from 300 μM to 0.1 µM, in a 384-well transparent plate (Greiner Bio-One, Madrid, Spain) with DMSO:PBS (1:99 vol/vol). After 2 h of incubation at 37°C, the plate was read in a NEPHELOstar Plus nephelometer (BMG LABTECH GmbH, Ortenberg, Germany) at 635 nm. The results were adjusted to a segmented regression to obtain the maximum concentration at which the compounds were soluble.

### Human plasma stability assay

Plates (96-well Polypropylene Deep Well Plate; Corning Inc., Somerville, MA, US) containing 100 µL of 5 µM compounds in human plasma (Seralab, Granollers, Spain), pooled from healthy donors and extracted in citrate tubes, were incubated at 37°C for different times (0, 60, 120, and 360 min). Then, 300 µL ACN was added to precipitate plasma protein, and the plate was centrifuged at 4000 × *g* for 60 min at 4°C. For sample quantification, the resulting supernatant was taken and analyzed by ultra performance liquid chromatography with tandem mass spectrometry (UPLC-MS/MS; ACQUITY Xevo TQD UPLC QSM; Waters) in an ACQUITY BEH C18 column (1.7 µm, 2.1 × 50 mm; Waters). As mobile phase (0.6 mL/min flow rate) was used the following gradient of 0.1% formic acid in water (A) or in ACN (B): 95% A:5% B from 0 to 0.1 min, gradual increase to 100% B from 0.1 to 1 min, maintenance in 100% B from 1 to 2 min, return to 95% A:5% B from 2 to 2.1 min, and maintenance of this ratio from 2.1 to 2.5 min. Compound concentrations were calculated from the MS peak areas.

### Human hepatocyte stability assay

A vial of human hepatocytes (100 donor pool HCP100.H15/Lot: 2010012; XenoTech, Kansas City, KS, US) was removed from the liquid nitrogen tank and immersed into a water bath. When only a small ice crystal remained, the cell suspension was transferred to a tube with the preheated suspension medium (OptiThaw hepatocyte medium, K8000; XenoTech). After a brief centrifugation at 100 × *g* for 5 min, hepatocytes were gently resuspended with incubation medium (OptiIncubate hepatocyte medium, K8400; XenoTech) and counted, and viability was determined using trypan blue staining. One-micromolar compounds were incubated at 37°C and 5% CO_2_ in the presence of 0.5 × 10^6^ hepatocytes in a 500-µL incubation volume. Aliquots were taken, and reactions were terminated with ACN at each of the six sampling time points (0, 15, 30, 60, 90, and 120 min). The samples were centrifuged, and the remaining compound was determined by UPLC-MS/MS analysis as above. Intrinsic clearance was calculated from the logarithm of the remaining compound at each of the times evaluated.

### Human microsomal stability assay

The following components were added in a 96-well microplate in a final volume of 500 µL 50 mM Na/K phosphate buffer, pH 7.4 (stock solutions are indicated in parentheses, dissolved in the same buffer unless otherwise specified): 1 µM NADP (10 mM), 10 µM glucose 6-phosphate (100 mM), 1 µM glucose 6-phosphate dehydrogenase (40 U/mL, dissolved in 5 mM sodium citrate), 3 µM MgCl_2_·6 H_2_O (30 mM, dissolved in H_2_O), 0.8 mg protein/mL of human microsomes (10 mg/mL protein; XenoTech), and 1 µM of test compound (100 µM, dissolved in ACN). Plates were incubated at 37°C and 75 µL samples were taken at 0, 10, 20, 40, and 60 min, to which 75 µL ACN + internal standard (Rolipram) to inactivate the microsomes and 30 µL of 0.5% formic acid in H_2_O to improve the chromatographic conditions were added, and kept at 4°C. When all the samples were taken, the plate was centrifuged at 46,000 × *g* for 30 min at 15°C, and UPLC-MS/MS was performed as above, except for the time in 100% B from 1 to 2.5 min, the return to 95% A:5% B from 2.5 to 2.6 min, and the maintenance of this ratio from 2.6 to 3.0 min. Metabolic stability was calculated from the logarithm of the remaining compound at each of the times evaluated.

### Cytochrome inhibition activity assay

To screen the inhibition potential of the compounds using recombinant human cytochrome P450 enzymes (CYP1A2, CYP2C9, CYP2C19, and CYP2D6) and probe substrates with fluorescent detection, incubations were conducted in a 200-µL volume in 96-well microtiter plates (Costar #3915; Corning Inc.). Addition of cofactor-buffer mixture (0.2 M KH_2_PO_4_ buffer, 1.3 mM NADP, 3.3 mM MgCl_2_, 3.3 mM glucose-6-phosphate, and 0.4 U/mL glucose-6-phosphate dehydrogenase), previously diluted control Supersomes (insect cell control for Supersomes enzymes, baculovirus-insect cell-expressed; Corning Inc.), standard inhibitors (furafylline, tranylcypromine, sulfaphenazole, and quinidine), and test compounds was carried out by a Zephyr liquid handling station (Caliper Life Sciences, Hopkinton, MA, US). The plate was then pre-incubated at 37°C for 5 min, and the reaction was initiated by the addition of pre-warmed enzyme/substrate mix, which contained buffer (KH_2_PO_4_), cDNA-expressed P450 in insect cell microsomes, substrate (3-cyano-7-ethoxycoumarin for CYP1A2 and CYP2C19, 7-methoxy-4-(trifluoromethyl)coumarin for CYP2C9, and 3-[2-(N,N-diethyl-N-methylammonium)ethyl]-7-methoxy-4-methylcoumarin for CYP2D6) to obtain the final assay concentrations in a reaction volume of 200 µL. Reactions were terminated after a specific time for each cytochrome by the addition of stop solution (ACN:0.5 M tris-HCl, 80:20 vol/vol). Fluorescence in each well was measured using a fluorescence plate reader (EnVision 2104 multilabel plate reader; PerkinElmer, Waltham, MA, US), and percentage of inhibition was calculated.

### Caco-2 permeability assay

Caco-2 cells (ATCC under the license of Abcam Inc., doing business as NaviCyte Scientific, Berkeley, CA) cultured to confluency were trypsinized and seeded onto a filter transwell insert (high-throughput screening Transwell 96-well permeable supports; Corning Inc.) at a density of ~10,000 cells/well in Dulbecco’s modified Eagle’s medium (DMEM). Confluent cells were subcultured at passages 58–62 and grown in a humidified atmosphere of 5% CO_2_ at 37°C. Following an overnight attachment period (24 h after seeding), the cell medium was replaced every other day with fresh medium in both the apical and basolateral compartments. The cell monolayers were used for transport studies 21 days post-seeding, when monolayer integrity was checked by measuring the transepithelial electrical resistance (TEER). If TEER values were ≥500 Ω/cm^2^, the medium was removed and the cells were washed twice with pre-warmed (37°C) Hank’s Balanced Salt Solution (HBSS). Compound stock solutions were made in DMSO and further diluted in HBSS (1% final DMSO concentration). All compounds, including controls (colchicine, E3S), were tested at a final concentration of 5 to 10 µM. For A → B directional transport, the donor working solution was added to the apical compartment (A) and HBSS as receiver working solution to the basolateral compartment (B). For B → A directional transport, the donor working solution was added to B and HBSS as receiver working solution to A. The cells were incubated at 37°C for 120 min with gentle stirring. At the end of the incubation, samples were taken from both donor and receiver compartments and transferred into 384-well plates, and compound concentrations were determined by UPLC-MS/MS analysis as for the plasma stability assay. After the assay, Lucifer Yellow (LY) was used to further validate the cell monolayer integrity; cells were incubated with 10 µM LY in HBSS for 1 h at 37°C, obtaining apparent permeability (Papp) values for LY of ≤10 nm/s, which confirmed the well-established Caco-2 monolayer.

### *L. infantum* cultures

*L. infantum* (MHOM/ES/2016/CATB101) promastigotes were maintained at 26°C in complete Schneider’s medium (Schneider’s insect medium supplemented with 10% FBS, 25 µg/mL gentamycin, 1% penicillin-streptomycin, and 1% sterile human urine, pH 6.7). For growth inhibition assays, promastigotes were used in the logarithmic growth phase, whereas for the infection of macrophages and generation of amastigotes they were used in the stationary phase. Axenic amastigotes were obtained following established protocols (40, 57–59); briefly, 1 × 10^7^ promastigotes/mL in stationary phase were cultured in Schneider’s insect medium supplemented with 20% FBS, 25 µg/mL gentamycin, and 3.9 g/L of 2-(*N*-morpholino)ethanesulfonic acid, at pH 5.4 and 37°C. After 48 h, the typical rounded morphology of axenic amastigotes and their flagellum shortening could be observed. This culture was used within a week after its preparation.

### Protein aggregation assays in *L. infantum* cultures

Ten milliliters of *L. infantum* promastigote cultures in the logarithmic growth phase at a concentration of 10^7^ cells/mL in T-25 flasks (SPL Life Sciences, Pochon, Kyonggi-do, South Korea) was either treated with 0.15 µM or 0.3 µM YAT2150 or left untreated. After 90 min and 4 h of incubation, 1 mL of each culture was washed (3×, 1 mL PBS, 600 × *g*, 3 min) and the parasite-containing pellets were taken up in 100 µL of 4.5 mg/mL NaCl supplemented with 1× cOmplete protease inhibitor cocktail (Roche, Basel, Switzerland) and incubated overnight at 4°C with gentle stirring. After this time, samples were spun down (2,000 × *g*, 10 s) and the protein in each supernatant was quantified with the Pierce BCA protein assay kit (Thermo Electron Corporation, Waltham, MA, US). In a 96-well flat bottom black plate (Greiner Bio-One), 2 µg of protein from each supernatant was diluted in a final volume of 100 µL PBS in duplicates. Protein aggregation was measured by the addition of 25 µM ThT to each well, and after a 15-min incubation with gentle stirring, ThT fluorescence emission intensity was measured as detailed above.

### Isolation of aggregation-prone proteins from *L. infantum* cultures

Proteins insoluble in 0.1% SDS were isolated following previously described protocols (60). First, 40 mL of a *L. infantum* preparation containing approximately 10^8^ *L. infantum* promastigotes in the logarithmic growth phase was spun down (50 × *g*, 3 min) to remove dead parasites and washed twice with sterile PBS supplemented with one tablet of cOmplete protease inhibitor cocktail in 10 mL. After centrifugation (600 × *g*, 3 min) to pellet the parasites, they were taken up in 300 µL of RIPA buffer (150 mM NaCl, 1% Triton X-100, 0.1% SDS, 2 mM EDTA, 5% glycerol, 50 mM tris-HCl, pH 9.4) supplemented with 1× cOmplete. The solution was homogenized in a bath sonicator (FB15053 ultrasonic bath) for six cycles (pulse: 30 s on, 30 s off) and incubated for 90 min at 4°C. Next, the lysate was spun (300 × *g*, 2.5 min, 4°C) to remove debris and unbroken cells and the supernatant was carefully loaded on top of 1 mL of 40% sucrose and ultracentrifuged (200,000 × *g*, 1 h) in order to pellet large insoluble aggregates. These were resuspended in 400 µL of lysis buffer (PBS containing 2% SDS, 5 mM DTT, and 2 mM EDTA; supplemented with 1× cOmplete) and incubated at 37°C for 30 min, pipetting up and down every 2 min with siliconized pipette tips. The resulting sample was spun down (16,000 × *g*, 12 min), and the supernatant was recovered and concentrated using an Amicon ultra 0.5 mL centrifugal filter, 3 kDa cutoff (Sigma-Aldrich Corporation). The protein in the concentrated solution was quantified using the Pierce BCA protein assay kit, and 100 µg of protein was loaded in a 12.5% SDS-PAGE stained with Coomassie Brilliant Blue R-250. The material not entering the resolving gel was excised and subjected to liquid chromatography with tandem mass spectrometry (LC-MS/MS) analysis.

### LC-MS/MS analysis

Trypsin digestion of proteins in gel slabs was performed in a ProGest automatic digestor (Genomic Solutions Inc., Ann Arbor, MI, US). Each sample was reduced (20 mM DTT in 25 mM NH_4_HCO_3_, pH 8.0, 60 min, 60°C), alkylated (55 mM iodoacetamide in 50 mM NH_4_HCO_3_, pH 8.0, 30 min, 25°C, protected from light), and digested for 2 h with 80 ng of porcine trypsin (sequencing-grade modified Trypsin Gold; Promega, Madison, WI, US) in 50 mM NH_4_HCO_3_, pH 8.0, 37°C. Another aliquot of enzyme was added, and the digestion was allowed to continue overnight under the same conditions. The resulting peptide mixture was extracted from the gel matrix with 5% FA in 50% ACN followed by 100% ACN, cleaned with a C18 tip (PolyLC Inc., Columbia, MD, US) as per manufacturer’s protocol, and finally dried in a SpeedVac concentrator and stored at ‒20°C until LC-MS/MS analysis. To increase peptide amounts, a third digestion with Proteinase K (Sigma-Aldrich Corporation) was performed. To both the extracted tryptic digest and the remaining gel band were added 100 ng Proteinase K in 500 mM NH_4_HCO_3_, pH 8.0, and after a 15 min digestion the resulting peptides were extracted from the gel as described above, pooled with the remaining in-solution digestion, dried in a SpeedVac concentrator and stored at ‒20°C until LC-MS/MS analysis.

Mass spectrometry was performed in a NanoAcquity HPLC system (Waters) coupled to an LTQ-OrbitrapVelos mass spectrometer (Thermo Fisher Scientific Inc.). The dried tryptic digests were taken up in 1% FA, and an aliquot was injected into the liquid chromatography system. Peptides were trapped in a Symmetry C18 trap column (5 µm, 180 µm × 20 mm; Waters) and separated in a C18 reverse-phase NanoAcquity UPLC BEH capillary column (130 Å, 1.7 µm, 75 µm × 250 mm; Waters), with a mobile-phase 1% to 40% B gradient in 30 min followed by a 40% to 60% B gradient in 5 min (A: 0.1% FA in water; B: 0.1% FA in ACN) and a flow rate of 250 nL/min. Eluted peptides were ionized in an emitter needle (PicoTip; New Objective Inc., Littleton, MA, US) with an applied spray voltage of 2 KV. A 300–1,600 *m/z* range of peptide masses was analyzed in a data-dependent mode where a full scan MS was acquired in the Orbitrap with a resolution of 60,000 full width at half maximum at 400 *m/z*. Within this range, the 15 most abundant peptides (≥500 counts) were selected from each scan and fragmented in the linear ion trap using collision-induced dissociation (38% normalized collision energy) with He as the collision gas. The scan time settings were as follows: full MS: 250 ms (1 microscan) and MS^n^: 120 ms. Generated *.raw data files were collected with Thermo Xcalibur (v. 2.2).

A database was created by merging all protein entries present in the public UniProt database for *L. infantum* (uniprot_Linfantum_taxonomy_5671_cont, v25/3/21) with a small database containing laboratory contaminant proteins. The *.raw data files obtained in the LC-MS/MS analyses were used to search with the SequestHT search engine using Thermo Proteome Discover (v1.4.1.14) against the aforementioned database. Both target and decoy databases were searched to obtain a false discovery rate (FDR) and thus estimate the number of incorrect peptide-spectrum matches that exceeded a given threshold, applying preestablished search parameters [enzyme: trypsin (semi); missed cleavage: 2; fixed modifications: carbamidomethyl of cysteine; variable modifications: oxidation of methionine and deamination of asparagine and glutamine; and peptide tolerance: 10 ppm and 0.6 Da for MS and MS/MS spectra, respectively]. To improve the sensitivity of the database search, the semi-supervised learning machine Percolator was used in order to discriminate correct from incorrect peptide spectrum matches. The Percolator assigns a *q*-value to each spectrum, which is defined as the minimal FDR at which the identification is deemed correct (0.01, strict; 0.05, relaxed). These *q*-values are estimated using the distribution of scores from decoy database search. Only proteins identified with at least two peptides (FDR ≤ 5%) are reported.

### Promastigote growth inhibition assay

In 96-well microtiter plates (Nunclon Delta surface; Thermo Fisher Scientific Inc.), serial dilutions (1:2) of the drugs or peptides were performed, either free or loaded in nanoformulations, in 100 µL of complete Schneider’s medium, to which one volume of 2 × 10^6^ logarithmic growth-phase *L. infantum* promastigotes/mL in the same buffer was added. After 48 h, resazurin sodium salt was incorporated at a final concentration of 0.00125% and the plates were incubated at 26°C for another 24 h, when fluorescence from resorufin was measured as described above.

### Amastigote growth inhibition assay

Compound activity on amastigotes was determined following the parasite rescue and transformation assay (61). RAW 264.7 macrophages were seeded in 96-well microtiter plates at 10^5^ cells/mL of DMEM supplemented with 10% FBS and 1% penicillin-streptomycin (complete DMEM, DMEMc) in a final volume of 200 µL per well. After an overnight incubation (37°C, 5% CO_2_) to allow cell adherence, the medium was removed and 10^6^ *L. infantum* promastigotes/mL were added (1:10 macrophage:promastigote ratio) in DMEM supplemented with 2% FBS and 1% penicillin-streptomycin (DMEM 2% FBS). After 24 h, parasites not internalized by macrophages were removed by washing (3×, PBS) and dilutions in DMEM 2% FBS of free or liposome-encapsulated compounds were added. After 48 h, the medium was removed, cells were washed three times with PBS, and 40 µL of 0.05% SDS in complete Schneider’s medium was added; after 40 s, 160 additional µL/well of complete Schneider’s medium was added and plates were incubated at 26°C for another 48 h. Then, resazurin sodium salt was incorporated at a final concentration of 0.00125% and plates were incubated for 24 h more in the same conditions. Afterwards, resorufin fluorescence was measured as above.

### Assessment of variation of ATP levels in *L. major*

The process described in reference (62) was followed. Briefly, *L. major* promastigotes (Friendlin strain) were transfected with the pLEXSY-hyg2.1 expression vector (Jena Bioscience, Jena, Germany) containing a cytoplasmic form of firefly luciferase mutated at its C-terminal tripeptide to prevent its import into the glycosome. Promastigotes were grown in Roswell Park Memorial Institute 1640 medium (RPMI) supplemented with 10% FBS, 2 mM L-glutamine, 1,000 U/mL penicillin/streptomycin (Gibco, Billings, MT, US), and 100 µg/mL hygromycin (InvivoGen, San Diego, CA, US) at 26°C. To assess variation in the intracellular concentration of free ATP, parasites were harvested at the late exponential phase of growth. After two washes with Hanks balanced buffer, parasites were resuspended in the same buffer supplemented with 10 mM D-glucose at 2.2 × 10^7^ parasites/mL. After the addition of DMNPE-caged D-luciferin (GoldBio, St. Louis, MO, US) at 50 µM final concentration, the parasite suspension was aliquoted (90 µL/well) into a black 96-microwell plate (Nunc A/S, Roskilde, Denmark), and luminescence readout was monitored in a POLARstar Galaxy microplate reader (BMG LABTECH GmbH) with a luminescence setup. Once the luminescence reached a plateau, 10 µL of a YAT2150 solution at 10× fold its final concentration was added (*t*_0_) and the luminescence value at this point was considered as 100%. DMSO in the parasite suspension was never above 1%. Controls for membrane permeabilization (0.1% Triton X-100) and inhibition of oxidative phosphorylation (1.5 µM 1,4-naphthoquinone) were used as controls, as well as untreated parasites. Experiments were made in duplicate and repeated at least twice.

### Flow cytometry

For the analysis of peptide targeting to promastigotes, 2.5 × 10^6^ promastigotes of the *L. infantum* MHOM/ES/2016/CATB101 strain in the logarithmic growth phase were placed in Eppendorf tubes in a volume of 500 µL of complete Schneider’s medium. Disaggregated Flu-AISVFFLEP-amide and Flu-DNFIFGQ-amide were reconstituted with complete Schneider’s medium and added to the cells in a final concentration of 50 µM. After overnight incubation, promastigotes were stained for 30 min with 4 µg/mL Hoechst 33342, washed with PBS (3×, 600 × *g*, 3 min), and finally fixed with 3% paraformaldehyde for 20 min. After three more PBS washes, the samples were diluted 1:5 in a final volume of 500 µL PBS in 5 mL polystyrene round-bottom tubes (Corning Inc.) and processed using a 20-parameter standard configuration in a five-laser LSRFortessa flow cytometer (BD Biosciences, San Jose, CA, US). Side- and forward-scatter were used in a logarithmic scale to determine the cell population, acquiring 10,000 events for each sample. Hoechst 33342 and fluorescein were detected by excitation with 350- and 488-nm lasers, and emission was collected with 450/50-BP and 525/50-BP filters, respectively.

For the analysis of YAT2150 targeting, 5 × 10^6^ promastigotes of the *L. infantum* MHOM/ES/2016/CATB101 strain in the logarithmic growth phase and 5 × 10^6^ axenic amastigotes from the same strain in day 5 of growth were placed in Eppendorf tubes with a final volume of 1 mL of complete Schneider’s medium. YAT2150 dissolved in DMSO was added in a final concentration of 0.38 µM (final DMSO concentration < 0.1%) along with Hoechst 33342 (4 μg/mL final concentration), incubated for 30 min, and washed (PBS, 300 × *g*, 3 min). Then, parasites were fixed and analyzed by flow cytometry as specified above, substituting fluorescein detection by YAT2150 detection (excitation with a 561-nm laser and emission collection with a 600LP-610/20-BP filter).

For the detection of antibody targeting to promastigotes, 1 × 10^7^ promastigotes of the *L. infantum* MHOM/ES/2016/CATB101 strain in complete Schneider’s medium were fixed in an Eppendorf tube for 20 min with 3% paraformaldehyde, washed 3× with PBS, and incubated for 1 h at room temperature with the anti-LPG monoclonal IgM antibody CA7AE diluted 1:500 in PBS containing 0.3% BSA. After three PBS washes, the cells were incubated for 1 h with a secondary goat anti-mouse antibody conjugated to Alexa Fluor 488 (AF488; Invitrogen, Waltham, MA, US) diluted 1:200 in PBS containing 2% BSA. After three final PBS washes, flow cytometry analysis was performed as specified above for peptide targeting with fluorescein, except for the acquisition of 30,000 events.

### Fluorescence microscopy

All the samples were visualized in an 8-well chamber slide (ibidi GmbH, Gräfelfing, Germany). For staining with the ProteoStat aggresome detection kit (Enzo Life Sciences Inc., Farmingdale, NY, US), *L. infantum* promastigotes and axenic amastigotes in days 3 and 5 of growth, respectively, were washed twice with PBS. In PBS, a 1:2,000 ProteoStat dye dilution was then added and after 5 min, 4 µg/mL Hoechst 33342 was incorporated and incubated for a further 10 min. The samples were then washed (3×, PBS) and observed with a Leica TCS SP5 laser scanning confocal fluorescence microscope. ProteoStat and Hoechst 33342 were detected by excitation through 561- and 405-nm lasers, respectively. Emission was collected between 590 and 670 nm for ProteoStat and between 415 and 500 nm for Hoechst 33342. The same protocol was followed for YAT2150 staining of promastigotes and axenic amastigotes, substituting ProteoStat by an incubation in 0.38 µM YAT2150. To quantify Manders’ overlap coefficient (63), images were analyzed using the Just Another Colocalization Plugin [JACoP (64)] in the Fiji software (65). Manders’ coefficient ranges from 0 to 1 showing the pixel percentage that is overlapped, being 0 defined as no colocalization and 1 as total colocalization.

For the analysis of peptide targeting to promastigotes, 200 µL of the samples prepared as described above in the flow cytometry section was placed in an 8-well chamber slide and fluorescence microscopy was conducted in a Zeiss LSM 800 equipment (Zeiss, Oberkochen, Germany) with a 100×/1.4 oil DIC M27 objective. Hoechst 33342 was excited with a 405-nm diode laser, 5 mW, class 3B, and fluorescein with a 488-nm diode laser, 10 mW, class 3B; emission was collected in the 390–460- and 500–700-nm ranges, respectively. A Z-stack image of 30 layers of a promastigote incubated with the Flu-AISVFFLEP-amide peptide was acquired in a Leica TCS SP5 laser scanning confocal fluorescence microscope, where Hoechst 33342 and fluorescein were excited with 405- and 488-nm diode lasers and emission was collected in the 414–474- and 501–562-nm ranges, respectively.

For the targeting analysis in promastigotes of YAT2150 encapsulated in CF-PE-containing liposomes, 10^7^ *L. infantum* promastigotes/mL were incubated in complete Schneider’s medium for 1 h with 0.38 µM YAT2150 contained in the liposome suspension, during the last 30 min in the presence of 4 µg/mL Hoechst 33342. Cells were then washed three times with PBS, fixed with 3% paraformaldehyde for 20 min, washed again, and visualized with a Leica TCS SPE laser scanning confocal fluorescence microscope. Hoechst 33342, CF-PE, and YAT2150 were excited with 405-, 488-, and 532-nm solid-state lasers and the respective emissions were collected in the 408–521-, 491–564-, and 589–691-nm ranges.

For the analysis of macrophages treated with YAT2150-stained promastigotes, RAW 264.7 macrophages maintained in DMEMc at 37°C and in the presence of 5% CO_2_ were seeded at 10^5^ cells/mL in DMEMc in an 8-well chamber slide, placing 300 µL per well and allowing cells to adhere overnight. In parallel, 10^7^ *L. infantum* stationary-phase promastigotes in 1 mL of DMEM 2% FBS were stained with 0.38 µM YAT2150 for 1 h at room temperature and thoroughly washed with DMEM 2% FBS (3×, 600 × *g*, 3 min). Then, RAW 264.7 cells were washed once with DMEM 2% FBS and 10^6^ YAT2150-stained *L. infantum* promastigotes/mL were added to each well and incubated for 24 h. Afterwards, 2 µg/mL Hoechst 33342 and 5 µg/mL wheat germ agglutinin-Oregon Green 488 (Thermo Fisher Scientific Inc.) were added simultaneously and incubated for 10 min, and the samples were visualized with a Leica TCS SP5 laser scanning confocal fluorescence microscope. Hoechst 33342, Oregon Green 488, and YAT2150 were excited with 405-, 488-, and 532-nm solid-state lasers and their respective emissions were collected in the 408–521-, 491–537-, and 605–707-nm ranges. For the targeting analysis in RAW 264.7 macrophages exposed to *Leishmania* of YAT2150 encapsulated in CF-PE-containing liposomes, the cell samples were prepared as described above, except for the use of non-stained promastigotes. *Leishmania*-exposed macrophages were incubated in DMEM 2% FBS for 3 h with 0.38 µM YAT2150 contained in the liposome suspension, during the last 10 min in the presence of 2 µg/mL Hoechst 33342. Then the cells were washed and observed as described above for promastigote targeting analysis.

For the observation of intracellular amastigotes, 6 × 10^7^ stationary-phase promastigotes were stained in 1 mL of cold PBS with 2.8 µg/mL of CFSE, incubated for 10 min (37°C, 5% CO_2_), and washed with PBS (3×, 600 × *g*, 3 min). The final CFSE-stained promastigote pellet was taken up in DMEMc and added to 5 × 10^5^ RAW 264.7 macrophages/mL that had been seeded in DMEMc and allowed to adhere overnight (10:1 promastigote:macrophage ratio). After 4 h of incubation, promastigotes were removed by washing five times with DMEM and cells were incubated for 10 min with 2 µg/mL of Hoechst 33342. Then, 0.38 µM YAT2150 was added, and after 6 h of incubation, the preparations were observed in an IX-51 Olympus (Tokyo, Japan) fluorescence microscope with a 100× objective. The fluorescence of Hoechst 33342, CFSE-stained parasites, and YAT2150 was detected with the fluorescence filter cubes U-MNU2, U-MWIBA3, and U-MWG2, which have excitation filters of 360–370, 460–495, and 534–588 nm and emission filters of 420, 510–550 and 609–683 nm, respectively.

For immunofluorescence microscopy analysis of the binding of anti-LPG monoclonal IgM antibody CA7AE to live promastigotes, the cells were treated as described above for flow cytometry except for the fixation step, which was omitted, and the staining, after removal by washing in complete Schneider’s medium of primary and secondary antibodies, with 4 µg/mL of the DNA dye Hoechst 33342 for 30 min. After three additional washes (600 × *g*, 3 min), 300 µL of a suspension of stained and washed promastigotes in complete Schneider’s medium was visualized with an IX-51 Olympus microscope as described above, using for AF488 the CFSE settings. For anti-LPG targeting analysis to live *Leishmania*-exposed and control non-treated macrophages, 5 × 10^4^ RAW 264.7 macrophages/mL were seeded in 300 µL/well of RPMI supplemented with 10% FBS and 1% penicillin-streptomycin in an 8-well chamber slide, and allowed to adhere for 24 h at 37°C in the presence of 5% CO_2_. *Leishmania*-treated macrophages were infected by incubation for 24 h in the presence of 5 × 10^5^ promastigotes of the *L. infantum* MHOM/ES/2016/CATB101 strain. After 3× PBS washes, the cells were prepared for immunocytochemistry analysis as described above for promastigotes, but using 2 µg/mL Hoechst 33342. Confocal fluorescence microscopy was conducted as described above for the analysis of fluorescein-tagged peptides.

### Statistical analysis

Unless otherwise stated, experiments were performed in triplicate and the results are expressed as mean values ± standard error of the mean (SEM). The IC_50_ and CC_50_ were calculated through non-linear regression analysis using GraphPad Prism 8.4 (GraphPad software, La Jolla, CA, US). Differences between samples were analyzed by one-way analysis of variance using the same software, considering a significant *P* value ≤0.05. Mean values and SEM were calculated using Microsoft Office Excel version 2306.

## SUPPLEMENTAL MATERIAL

Figs. S1 to S7, Tables S1 to S3 aac.01127-23-s0001.pdf Supplemental figures and tables.

ASM does not own the copyrights to Supplemental Material that may be linked to, or accessed through, an article. The authors have granted ASM a non-exclusive, world-wide license to publish the Supplemental Material files. Please contact the corresponding author directly for reuse.
