# Supplementary material for: Effect of the aggregated protein dye YAT2150 on Leishmania parasite viability
Source: Antimicrob Agents Chemother. 2024 Feb 13;68(3):e01127-23. doi: 10.1128/aac.01127-23 (PMC10916400; doi:10.1128/aac.01127-23)
Supplement: Figs. S1 to S7, Tables S1 to S3 — Supplemental figures and tables. [file aac.01127-23-s0001.pdf]

Effect of the aggregated protein dye YAT2150 on *Leishmania* parasite viability

Lucía Román-Álamo, Yunuen Avalos-Padilla, Inés Bouzón-Arnáiz, Valentín Iglesias, Jorge Fernández-Lajo, Juan M. Monteiro, Luis Rivas, Roser Fisa, Cristina Riera, David Andreu, Carlos Pintado-Grima, Salvador Ventura, Elsa M. Arce, Diego Muñoz-Torrero, Xavier Fernàndez-Busquets

Supplemental Material

Supplemental Figures and Tables

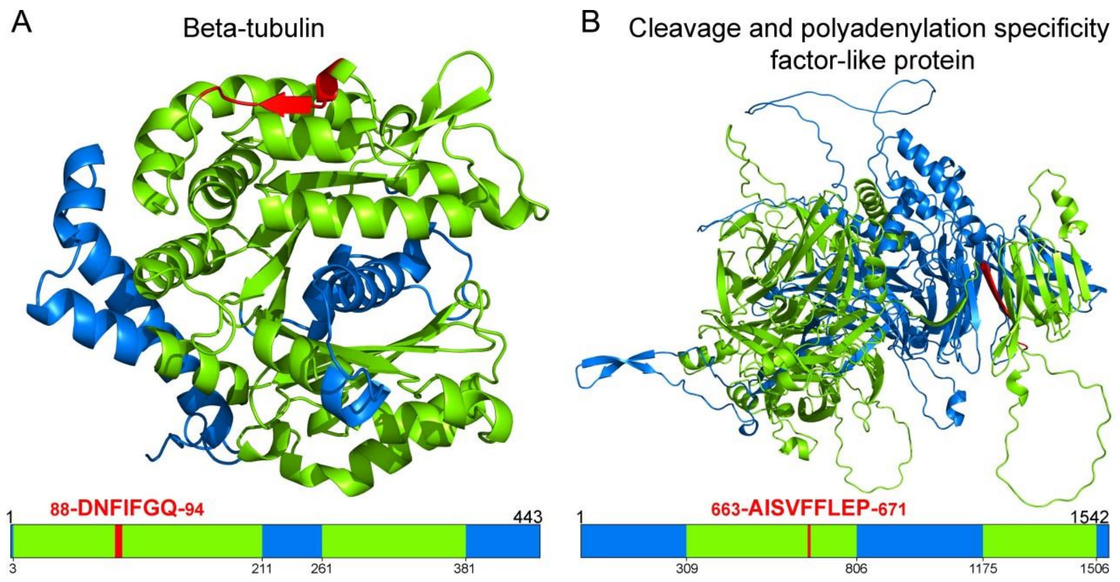

Fig. S1. Structural and sequential context of two amyloid peptides selected from the aggregation-prone *L. infantum* protein pool resisting dissolution in 0.1% SDS. Peptides from (A) β-tubulin and (B) cleavage and polyadenylation specificity factor-like protein (CPSF) (coloured red) are located inside "Tubulin" and "Mono-functional DNA-alkylating methyl methanesulfonate N-term" Pfam domains (coloured green). Non-identified globular domains are represented in blue. Protein models correspond to AlphaFold database version 3 (1).

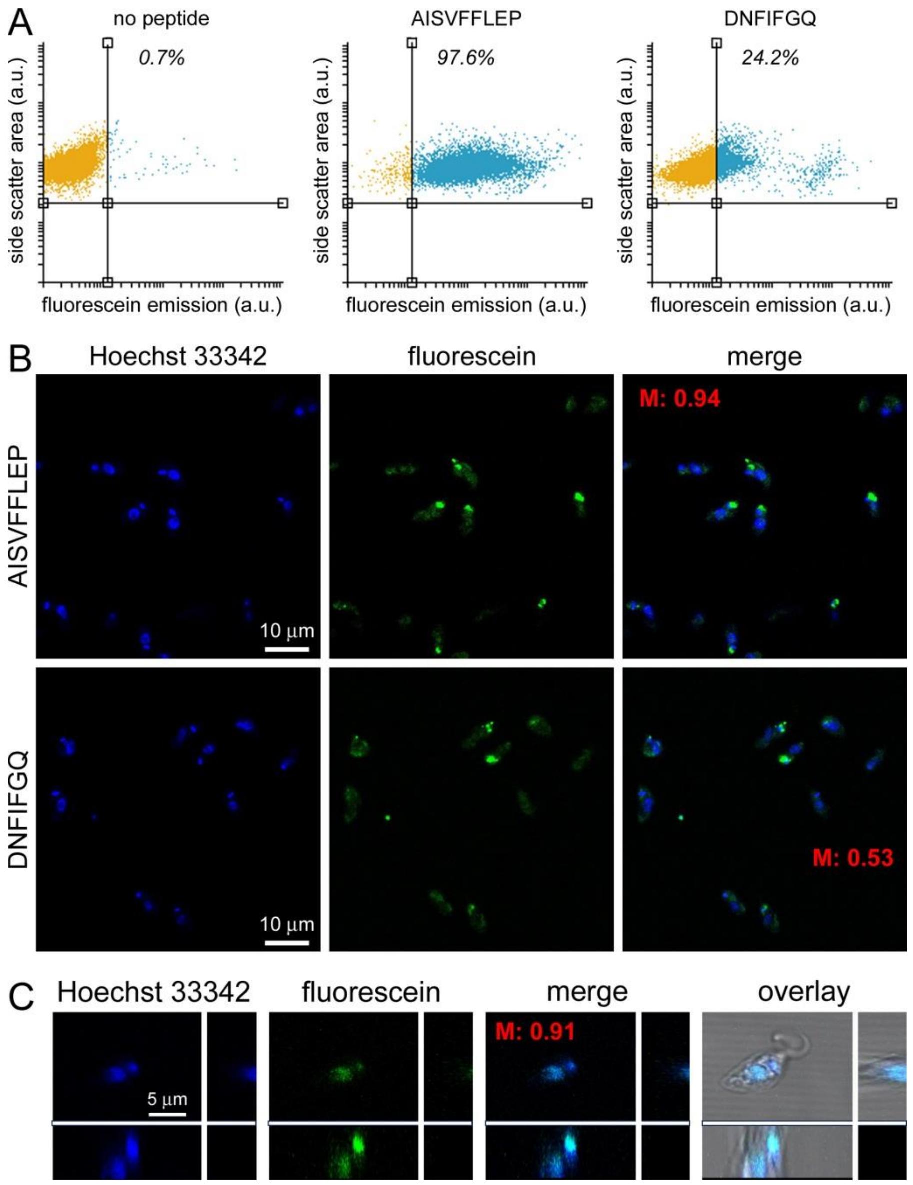

Fig. S2. Characterization of the interaction with *L. infantum* promastigotes of the fluorescein-labeled AISVFFLEP and DNFIFGQ peptides. (A) Flow cytometry analysis of promastigote targeting with 50 µM TFA-disaggregated fluorescein-labeled peptides after overnight coincubation. Percentages indicate the fraction of promastigotes with fluorescein signal above the set threshold. a.u.: arbitrary units. (B) Confocal fluorescence microscopy analysis of the intracellular location of 50 µM TFA-disaggregated fluorescein-labeled peptides after overnight coincubation with live promastigotes. (C) Z-stack image (30 layers) of AISVFFLEP viewed through orthogonal sections (XZ below and YZ on the right). M: Manders' correlation coefficients indicating the fraction of fluorescein-labeled peptide (green fluorescence) co-localized with Hoechst 33342 (blue fluorescence).

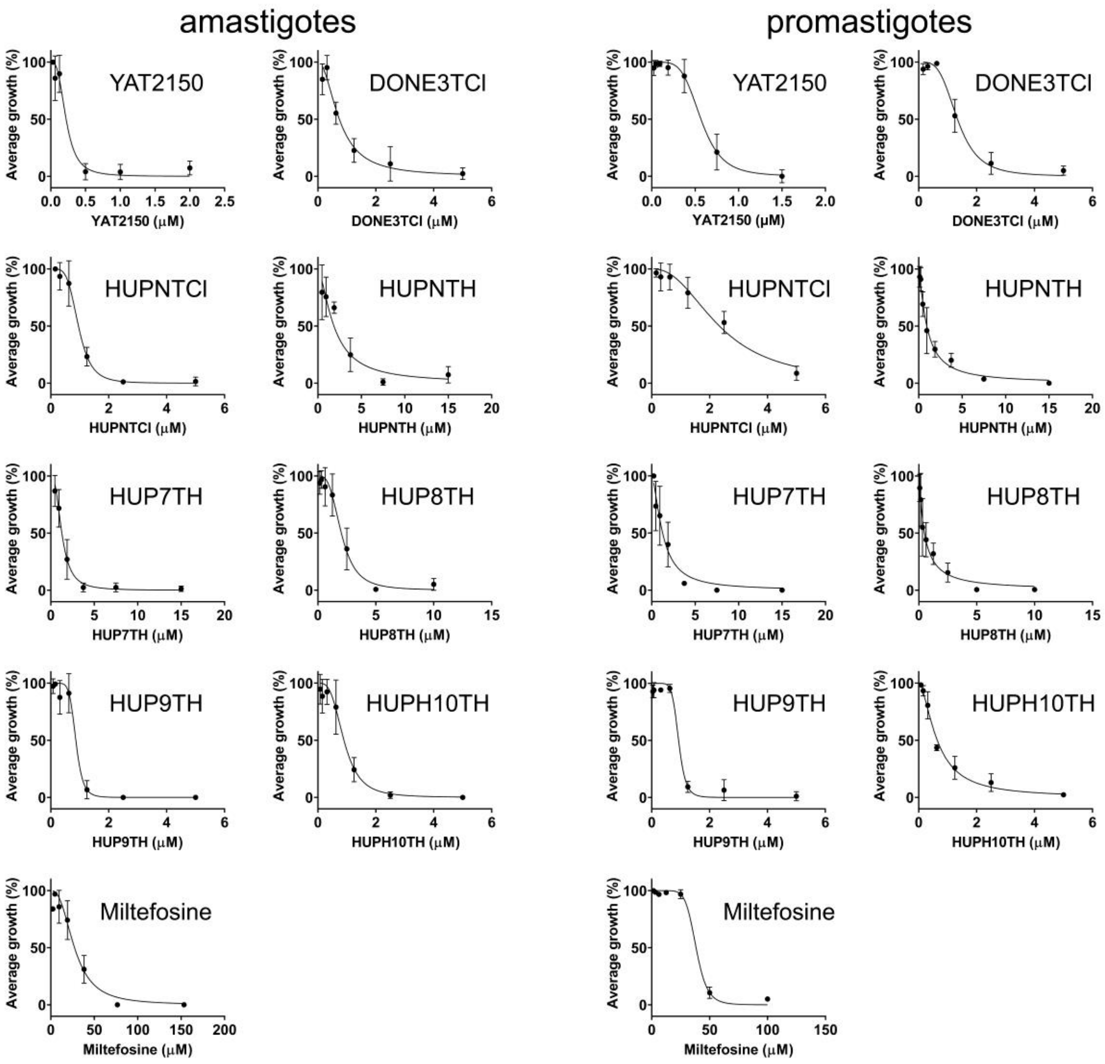

Fig. S3. Dose-response curves of the activity assays in *L. infantum* amastigotes and promastigotes for the compounds whose corresponding data are reported in Table 2.

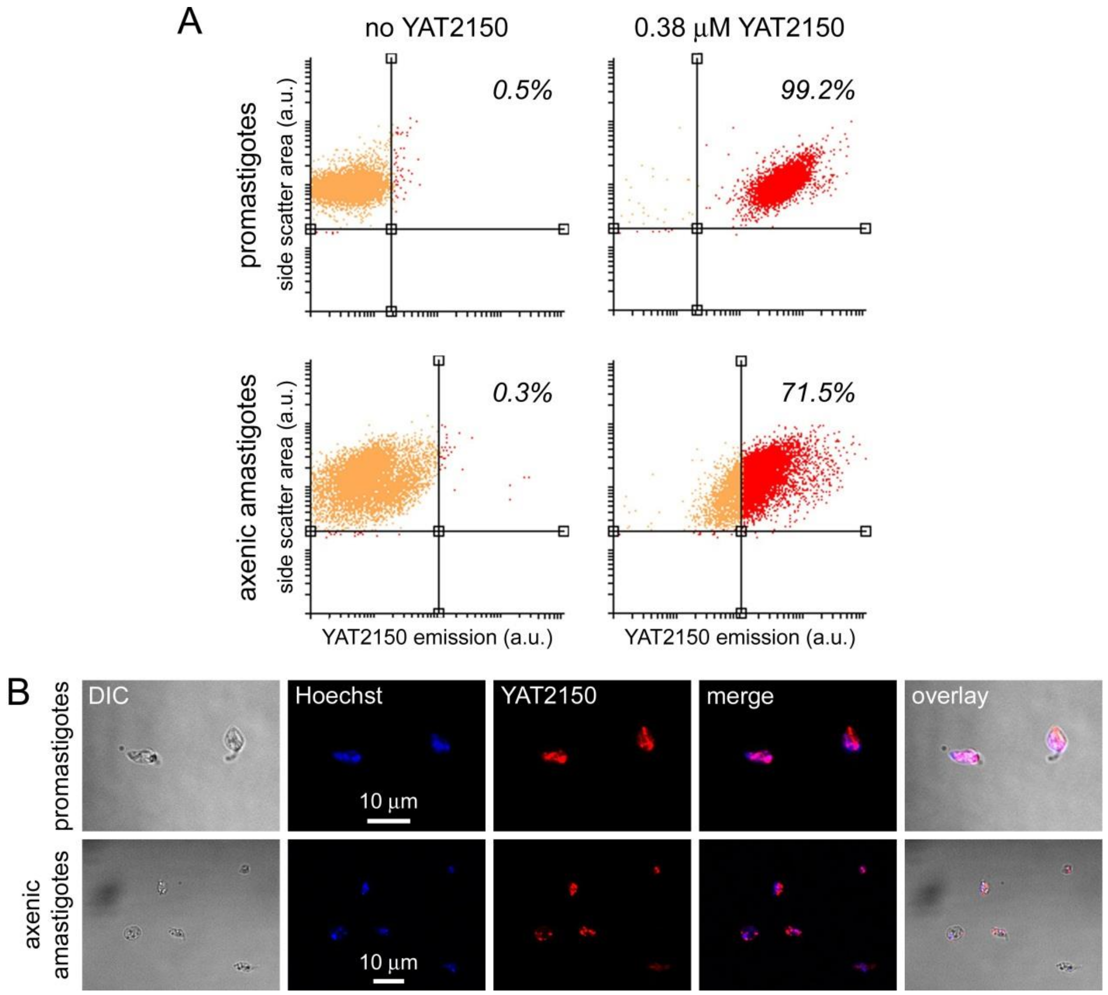

Fig. S4. Binding of YAT2150 to *L. infantum* promastigotes and axenic amastigotes. (A) Flow cytometry analysis of promastigotes and axenic amastigotes exposed for 30 min to 0.38 µM YAT2150. Percentages indicate the fraction of cells above the set YAT2150-positive threshold. a.u.: arbitrary units. (B) Detection by confocal fluorescence microscopy of intracellular protein aggregates in live promastigotes and axenic amastigotes following staining with 0.38 µM YAT2150 for 15 min. DIC: differential interference contrast image.

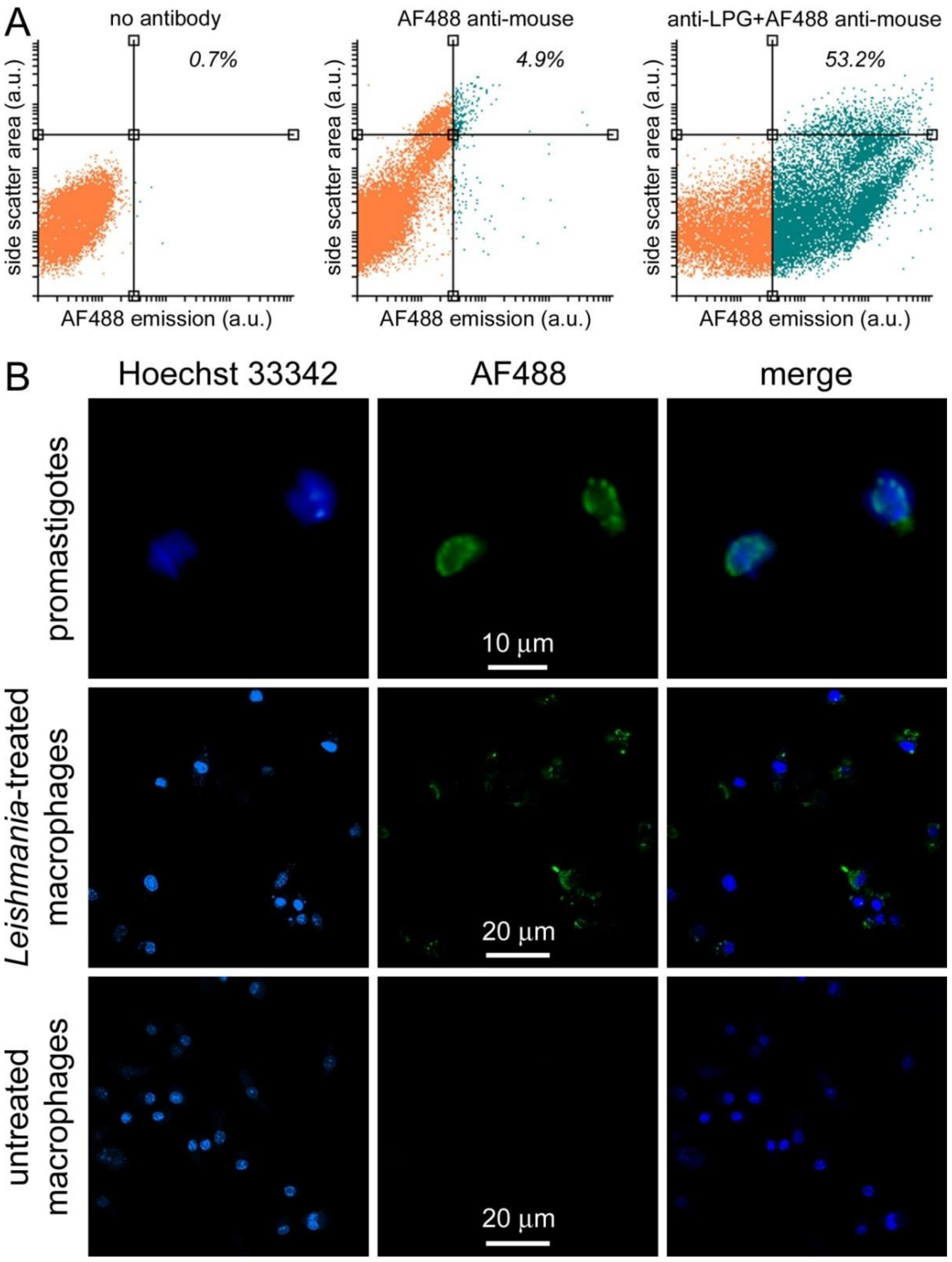

Fig. S5. Detection of *L. infantum* by the commercial anti-LPG monoclonal IgM antibody CA7AE. (A) Flow cytometry analysis of fixed promastigote targeting. Controls include an antibody-free promastigote preparation and a promastigote sample where the primary anti-LPG antibody had been omitted. Percentages indicate the fraction of promastigotes above the set LPG-positive threshold. a.u.: arbitrary units. (B) Fluorescence microscopy analysis of live promastigote and amastigote-infected macrophage targeting, including a control of macrophages not exposed to *Leishmania* and incubated with the anti-LPG antibody (untreated macrophages).

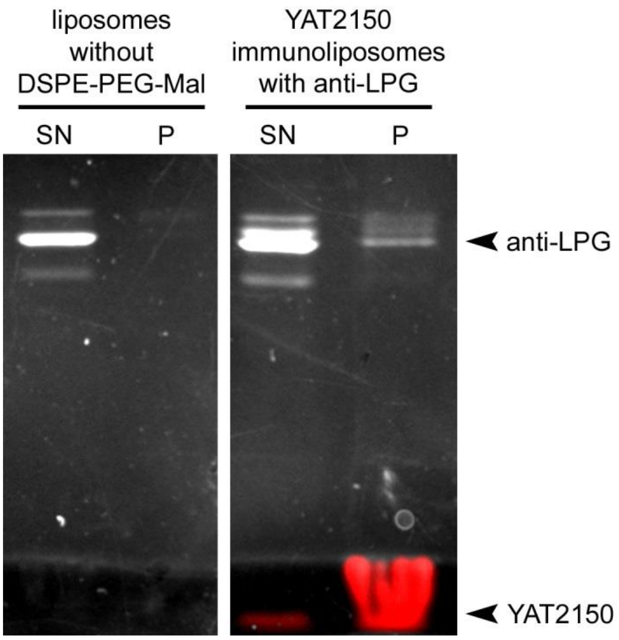

Fig. S6. Immunoliposome characterization. SDS-PAGE analysis of YAT2150-containing immunoliposomes functionalized with anti-LPG monoclonal antibody, compared to control liposomes lacking DSPE-PEG-Mal and subjected to the same experimental procedure. SN: supernatant after ultracentrifugation, P: pellet after ultracentrifugation, taken up in the same volume as the supernatant of PBS containing 10 mM EDTA.

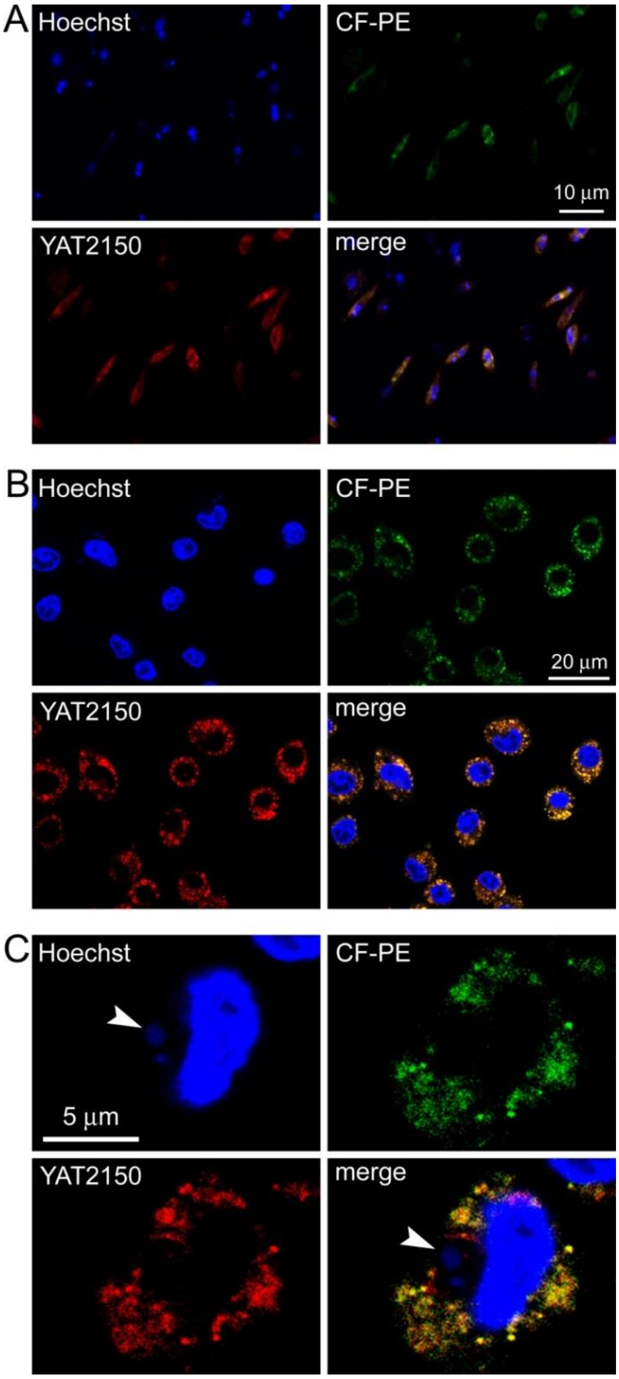

Fig. S7. Confocal fluorescence microscopy analysis of the cell targeting of 0.38 µM YAT2150 encapsulated in fluorescein-labeled liposomes. (A) Live *L. infantum* promastigotes were incubated for 1 h with liposomes, stained with Hoechst 33342, and fixed. (B,C) RAW 264.7 macrophages that had been exposed to *L. infantum* promastigotes were incubated for 3 h with liposomes, stained with Hoechst 33342, and fixed. The green fluorescence corresponds to CF-PE incorporated in the liposome formulation. The arrowhead indicates a *Leishmania* amastigote inside its phagolysosome.

Supplementary Table S1. *L. infantum* proteins insoluble in 0.1% SDS. Membrane proteins are shadowed in grey.

Accession	UniProtKB protein name	Score	Coverage	# Proteins	# Unique Peptides	# Peptides	# PSMs	# AAs	MW (kDa)	pI
A4I4B4	ATP-binding cassette protein subfamily A, member 10	293.68	31.99	1	62	62	128	1866	206.8	6.90
A0A381MFJ0	Plasma membrane ATPase	156.39	30.49	2	29	29	70	974	107.3	5.63
A0A381MS01	Tubulin β chain	128.56	44.47	2	18	18	50	443	49.7	4.82
A4I0B5	Hypothetical protein - conserved	127.54	35.43	1	21	21	52	556	62.5	6.99
A4HY43	ADP,ATP carrier protein 1, mitochondrial	116.48	48.58	1	15	15	51	317	35.1	9.70
A4HUE7	Putative folate / biopterin transporter	113.29	17.29	1	13	13	46	700	76.2	6.67
A4I6P8	H(+)-exporting diphosphatase	103.11	31.21	1	19	19	45	801	83.2	5.25
A0A6L0X265	Tb-292 membrane associated protein-like protein	85.01	46.77	2	23	23	38	2739	304.0	5.19
A4I1M5	Enoyl-CoA reductase, putative	84.65	36.27	1	15	15	36	306	34.1	9.51
A4IBA6	Calcium motif p-type ATPase, putative	80.06	25.70	1	20	20	33	1109	121.8	5.69
A4HRH5	Long-chain-fatty-acid-CoA ligase, putative	73.52	22.10	1	13	13	30	715	77.7	6.57
A4HW91	Hypothetical protein - conserved	71.39	18.99	1	15	15	28	1006	111.5	4.42
A4HSP6	Guanine nucleotide-binding protein subunit β-like protein	69.62	34.07	1	17	17	31	675	75.4	6.80
A0A381MCS3	Tubulin α chain	66.72	45.90	3	18	18	30	451	49.7	5.02
A4IAK2	Coatomer subunit α	65.15	18.98	1	20	20	27	1196	132.6	7.52
A4HW58	Putative kinesin K39	60.33	44.57	2	7	7	22	2926	326.2	4.48
A0A6L0XIU2	Nodulin-like, putative	52.37	19.79	2	9	9	22	672	73.7	7.61
A4I8D8	Putative ribosomal protein L3	50.56	26.97	1	10	10	21	419	47.5	11.05
A4IC24	TPR repeat / tetratricopeptide repeat / uncharacterized protein family (UPF0121)	49.96	31.41	1	10	10	20	382	42.7	9.19
E9AG72	Pteridine transporter (truncated), putative	49.91	20.83	1	10	10	21	653	71.0	7.99
E9AHC1	Putative cysteine peptidase, Clan CA, family C2	40.29	3.43	1	10	11	21	4343	489.5	5.66
A4I9R3	ATP-binding cassette protein subfamily B - member 2, putative	38.83	13.05	1	14	14	17	1341	146.5	6.28
A4HW10	Elongation of fatty acids protein	38.38	13.27	1	3	3	17	294	32.8	8.84
Q9NJS2	Reticulon-like protein	37.67	26.40	1	7	7	17	197	22.1	8.97
A4HWJ3	40S ribosomal protein S3, putative	36.36	42.01	1	8	8	19	219	24.5	9.79
A4HW09	Elongation of fatty acids protein	36.00	18.31	1	4	4	13	284	32.3	9.41
A4I9N9	Uncharacterized protein (fragment)	35.08	14.30	1	7	7	11	1322	144.4	4.70
A0A6L0WSB2	ATP-binding cassette protein subfamily G - member 1, putative	31.52	19.18	3	10	10	12	657	73.2	9.00
A4HW14	Elongation of fatty acids protein	30.91	31.27	1	8	9	15	323	37.2	9.07
A4IB09	Dolichyl-diphosphooligosaccharide-protein glycotransferase	29.83	11.61	4	2	9	13	784	86.9	9.00
A4I059	ATP-binding cassette protein subfamily C, member 1	28.55	8.03	1	2	9	11	1570	173.1	6.71
A4IC65	SPRY domain / HECT-domain (ubiquitin transferase)	28.33	2.22	1	12	12	13	6624	732.7	6.44
A0A6L0X791	Elongation factor 1-α	27.75	29.62	4	9	9	16	449	49.0	8.98
E9AHU4	Dolichyl-diphosphooligosaccharide-protein glycotransferase	26.34	11.60	1	2	9	12	836	92.2	8.59
A4I060	ATP-binding cassette protein subfamily C, member 2	25.49	8.90	1	2	9	11	1562	172.1	7.40
E9AHD7	Amino acid transporter aATP11, putative	22.70	10.60	1	5	5	9	500	55.4	6.54
A0A6L0XP99	Hypothetical protein - conserved	20.88	20.01	1	6	6	8	2804	317.0	4.79
A4I6E4	Cytoskeleton-associated protein CAP5.5, putative	20.27	12.43	1	8	8	9	724	79.9	5.68
A4HWK2	Tryparedoxin peroxidase	19.98	33.67	1	7	7	9	199	22.2	7.15
A4IDZ5	Mkiaa0324 protein-like protein	19.55	14.00	1	7	7	13	500	56.4	12.34
A4I9G8	Delta-12 fatty acid desaturase	19.04	12.94	1	4	4	9	394	45.4	8.03
A4HY18	NADH:ubiquinone oxidoreductase 78 kDa subunit-like protein	18.35	27.91	2	4	4	7	258	28.6	5.01
A4I9L8	Calcium channel protein, putative	18.29	3.01	1	6	6	8	2556	291.2	8.47
A4I8T4	Putative sterol C-24 reductase	18.20	13.10	1	5	5	10	496	58.1	9.14
A4HUD2	Amastin surface glycoprotein, putative	18.10	6.53	1	3	3	8	490	54.3	8.69
A0A6L0Y277	Polyubiquitin, putative	17.97	44.74	5	3	3	8	3040	341.0	7.77

E9AH65	UBA / TS-N domain containing protein, putative	17.76	14.14	1	4	4	6	396	42.9	10.35
A4HW18	Elongation of fatty acids protein	17.53	15.51	1	4	4	8	303	34.4	8.46
A4IAD3	Transmembrane 9 superfamily member	17.10	10.52	1	5	5	7	637	70.7	8.85
E9AGL0	Elongation of fatty acids protein	17.05	15.38	2	3	4	7	299	34.7	8.21
A4HVZ0	Amastin surface glycoprotein, putative	15.42	9.12	2	3	3	7	274	30.3	9.50
A4HWX5	N-terminal region of chorein - a TM vesicle-mediated sorter, putative	15.10	1.31	1	5	5	9	5661	605.6	8.18
A4I9G2	Coatomer subunit β	14.73	6.79	1	6	6	6	884	97.6	5.19
A4IBY3	Mitochondrial phosphate transporter, putative	14.53	24.92	1	7	7	8	317	34.6	9.16
A4I1J0	Protein of unknown function (DUF1295) / phospholipid methyltransferase, putative	14.38	8.56	1	3	3	7	257	28.8	9.86
A4HZQ0	Amino acid permease, putative	14.33	10.72	1	4	4	7	485	53.9	8.00
E9AHT2	Amastin-like protein	14.21	10.43	1	2	2	6	211	23.9	4.54
A4IB10	Dolichyl-diphosphooligosaccharide-protein glycotransferase	13.38	7.43	1	2	5	6	794	88.7	7.28
A0A381MN47	Histone H2A	13.18	25.76	3	3	3	5	132	13.9	10.96
E9AHB8	CDP-diacylglycerol-inositol 3-phosphatidyltransferase	13.04	32.16	1	5	5	7	227	25.3	7.83
A4HSI0	Hypothetical protein - conserved	12.77	6.68	1	3	3	4	779	86.4	7.64
A4I186	Guanine nucleotide-binding protein subunit β-like protein	12.70	6.00	1	4	4	4	1017	109.6	6.79
E9AHM8	Heat shock protein 83-1	12.68	7.43	2	4	4	5	686	78.7	5.21
A4I132	Hypothetical predicted multi-pass transmembrane protein	12.27	12.57	1	2	2	5	175	20.0	9.06
A4HTD0	Cation-transporting ATPase	12.24	8.12	1	7	7	9	1244	139.4	7.28
A4HSE7	Very-long-chain (3R)-3-hydroxyacyl-CoA dehydratase	12.20	22.77	1	4	4	5	224	25.0	9.63
A4I7B9	Phosphatidylethanolamine N-methyltransferase-like protein	11.75	4.81	1	3	3	6	582	66.7	7.94
A4HTE1	N-terminal region of chorein - a TM vesicle-mediated sorter, putative	11.30	2.64	1	5	5	5	2542	276.2	6.37
A4IA88	Lipophosphoglycan biosynthetic protein (Lpg2)	11.14	6.45	1	2	2	4	341	37.2	9.28
A4HW98	Histone H4	11.05	18.00	2	2	2	4	100	11.4	10.55
A4I7K4	ATP-dependent RNA helicase, putative	11.03	6.35	1	3	3	4	614	66.8	8.88
A0A381N054	Glucose transporter, lmGT2	10.68	5.11	1	2	2	4	567	61.2	6.25
A4IAI4	ER membrane protein complex subunit 1	10.42	6.01	1	3	3	6	815	87.0	6.38
E9AGW6	Intraflagellar transport protein 172, putative	10.18	4.83	1	5	5	6	1800	199.8	6.61
A4HYL7	Midasin	10.09	0.91	1	4	4	4	4825	534.6	5.31
A4HWJ9	NAD-specific glutamate dehydrogenase	9.94	6.47	1	5	5	5	1020	115.2	7.77
A4I020	Hypothetical protein - conserved	9.45	4.93	1	3	3	4	730	76.9	8.41
A4HX43	Putative kinesin	9.44	62.26	1	3	3	3	2811	304.9	4.21
A4I3I9	Amastin surface glycoprotein, putative	9.22	7.51	1	3	3	4	519	57.8	9.44
A4HYZ5	40S ribosomal protein S11, putative	9.11	15.60	1	2	2	5	141	16.3	10.89
A4HSH2	ATP synthase F1 - α subunit, putative	8.92	7.67	1	4	4	4	574	62.5	9.72
A4I7T3	Elongation of fatty acids protein	8.36	5.77	1	2	2	3	381	43.1	9.64
A4HWS5	Ankyrin repeats / α/β hydrolase family, putative	8.10	6.87	1	3	3	3	495	56.1	8.40
A0A381MCU2	40S ribosomal protein S4	8.07	7.33	2	2	2	3	273	30.6	10.29
A4I2N6	Calpain-like cysteine peptidase, putative	8.02	1.44	1	5	6	6	6168	701.1	5.33
A4HV14	Pretranslocation protein - α subunit, putative	7.88	12.35	1	4	4	4	486	53.9	8.87
A4IDG6	Inosine-guanosine transporter	7.81	7.41	1	3	3	3	499	54.0	6.89
Q9BHZ6	Elongation factor-1 gamma	7.74	5.45	3	2	2	3	404	46.2	5.49
A4HW07	Elongation of fatty acids protein	7.50	12.14	1	2	2	4	280	31.7	9.03
A4I6K0	Amino acid transporter aATP11, putative	7.31	4.50	1	2	2	3	511	55.9	7.88
A4I194	ABC transporter - mitochondrial, putative	7.23	5.95	1	3	3	4	656	71.6	8.82
A4I115	Transketolase	7.20	4.17	1	2	2	3	671	71.8	6.64

A4I7B1	Iron / zinc transporter protein-like protein	7.04	15.28	1	5	5	6	432	45.4	6.07
A4HTF0	Putative vacuolar-type Ca^{2+}-ATPase (fragment)	7.03	4.41	3	3	3	3	929	101.7	6.38
A4I4G7	Putative ribosomal protein L1a	7.03	7.51	2	3	3	3	373	41.1	11.60
A4HUB4	Putative ribosomal protein l35a	7.01	22.92	1	3	3	3	144	16.4	11.77
A0A499SND2	Amastin (fragment)	6.82	16.39	3	2	2	4	183	19.6	8.69
A4IBZ5	Hypothetical protein - conserved	6.77	2.03	1	3	3	3	1479	161.2	6.54
A0A6L0WK02	Microtubule-associated protein, putative	6.74	46.77	2	2	2	3	1732	195.4	4.78
A4I7V5	Cleavage and polyadenylation specificity factor-like protein	6.49	2.79	1	3	3	3	1542	167.3	6.62
A0A381MVV1	D-isomer specific 2-hydroxyacid dehydrogenase-like protein	6.43	7.16	1	2	2	2	335	36.8	5.63
A4HUB6	Dehydrogenase-like protein	6.31	5.83	1	2	2	2	412	44.9	8.46
A4HW08	Elongation of fatty acids protein	5.74	10.88	1	3	3	4	285	32.3	8.68
A4IE10	Hypothetical protein - conserved	5.73	4.01	1	2	2	2	623	70.2	8.43
A4HY08	Putative pyruvate dehydrogenase E1 component α subunit	5.67	6.08	1	2	2	2	378	42.7	8.18
A4ICH2	Dihydrolipoamide acetyltransferase component of pyruvate dehydrogenase complex	5.49	7.34	1	2	2	2	463	48.6	7.42
A4I4W0	60S ribosomal protein L13, putative	5.37	13.18	1	2	2	2	220	24.7	10.89
A4I4A1	Hypothetical protein - conserved	5.31	6.04	1	2	2	2	364	42.1	9.38
A0A6L0WLW0	Pteridine transporter (truncated), putative	5.31	3.46	1	2	2	2	722	78.9	5.49
A4I1D9	Dynein heavy chain, putative	5.19	1.06	1	3	3	3	4702	536.4	6.55
A4IAM6	Hypothetical protein - conserved	5.17	19.23	1	2	2	2	156	17.1	10.13
A4I5R7	Hypothetical protein - conserved	5.11	0.69	1	2	2	2	3790	408.7	6.39
A4I401	Cytochrome oxidase assembly protein-like protein	5.09	5.54	1	2	2	2	415	46.3	10.18
A4I4W5	ATP-dependent 6-phosphofructokinase	4.81	3.91	1	2	2	2	486	54.0	9.06
A4I0V7	Inhibitor of apoptosis-promoting Bax1, putative	4.78	10.54	1	2	2	3	313	33.1	9.83
A4I5B9	Amastin-like surface protein-like protein	4.49	9.46	1	2	2	2	222	24.5	6.89
A4I1H9	Hypothetical protein - conserved	4.39	0.77	1	2	2	2	2596	277.6	6.74
A4IAU0	40S ribosomal protein S3a-1	4.35	8.71	2	2	2	2	264	30.0	10.29
A4HT92	40S ribosomal protein S9, putative	4.00	8.95	1	2	2	2	190	22.1	10.65
A0A6L0XS88	Calpain-like cysteine peptidase, putative	3.91	16.66	3	2	2	5	4681	539.7	5.12
A4IBE6	*Chlamydia* CHLPS protein (DUF818) / α/β hydrolase family, putative	3.69	10.25	1	3	3	3	400	44.3	8.21
A0A381MBS1	Amino acid permease 24, putative	3.54	4.30	2	2	2	2	488	53.9	6.42
A4I948	ATP-binding cassette protein subfamily D, member 3	2.95	3.28	1	2	2	2	640	71.1	8.98
A4I116	40S ribosomal protein S8	2.90	10.91	1	2	2	2	220	24.9	11.33
A4HW62	Phosphopyruvate hydratase	2.77	6.99	1	2	2	2	429	46.0	5.45
A0A6L0XPU2	Short chain dehydrogenase, putative	2.69	7.69	2	2	2	2	273	29.9	9.06
A4I8A1	Hypothetical protein - conserved	2.57	1.62	1	2	2	2	1361	148.3	7.75
A4HVJ3	Palmitoyltransferase	2.44	4.28	1	2	2	2	607	65.8	5.31
A4HWI7	ATP-binding cassette protein subfamily G, member 4	2.38	4.72	1	2	2	4	741	83.0	6.61
A4I3D7	ER lumen protein-retaining receptor	2.23	15.98	1	2	2	3	219	26.0	9.39
A4HU00	Hypothetical protein - conserved	2.04	3.45	1	2	2	3	1247	132.8	7.59
A4I1Y3	GPN-loop GTPase 2	0.00	14.72	1	2	2	2	326	36.5	4.98

Accession: UniProtKB protein accession number.
Score: Sum of the scores of the individual peptides.
Coverage: Percentage of amino acids found in the analyzed peptides compared to the total number of amino acids in the entire sequence of the protein.
Proteins: The number of identified proteins in a protein group (all proteins that are identified by the same set of peptides).
Unique peptides: The number of peptide sequences unique to a protein group.
Peptides: The number of distinct peptide sequences in the protein group.
PSMs: The total number of identified peptide sequences for the protein.
AAs: The total number of amino acids of the entire sequence of the protein.
MW (kDa): The molecular weight of the protein calculated as the sum of the molecular weight of each amino acid without considering post-translational modifications.
pI: Protein isoelectric point.

Supplementary Table S2. GO enrichment analysis for the dataset of *L. infantum* proteins found in 0.1% SDS-resistant aggregates.

Category	Term	Count	%	*p*-value	Genes	List total	Pop hits	Pop total	Fold enrichment	Bonferroni	Benjamini	FDR
Biological process	Translation	18	19.35	5.19^{-10}	A4HT92, A4HWJ3, A4HYZ5, A4I4G7, A0A381MCU2, A4I116, A4HUB4, A4I4W0, A4IAU0, A4I8D8	34	170	1804	5.62	7.27^{-9}	7.27^{-9}	7.27^{-9}
Cell component	Nucleosome	6	6.45	2.60^{-4}	A4HW98	68	23	2531	9.71	0.004	0.004	0.004
	Ribosome	15	16.13	1.46^{-3}	A4I186, A4HYZ5, A4I4G7, A0A381MCU2, A4I116, A4HUB4, A4HSP6, A4I4W0, A4I8D8		221	2531	2.53	0.025	0.012	0.012
	Small ribosomal subunit	4	4.30	4.19^{-3}	A4HT92, A4HWJ3		13	2531	11.45	0.069	0.024	0.024
Molecular function	Structural constituent of ribosome	18	19.35	2.07^{-8}	A4HT92, A4HWJ3, A4HYZ5, A4I4G7, A0A381MCU2, A4I116, A4HUB4, A4I4W0, A4IAU0, A4I8D8	64	176	3190	5.10	8.5^{-7}	8.5^{-7}	8.5^{-7}
	Protein heterodimerization activity	6	6.45	1.00^{-4}	A4HW98		25	3190	11.96	4.1^{-3}	2.1^{-3}	2.1^{-3}
	ATPase activity, coupled to transmembrane movement of substances	4	4.30	4.60^{-2}	A4I4B4, A4I9R3, A4I948, A4I194		41	3190	4.86	8.5^{-1}	5.3^{-1}	5.3^{-1}
	rRNA binding	3	3.23	5.29^{-2}	A4HT92, A0A381MCU2		19	3190	7.87	8.9^{-1}	5.3^{-1}	5.3^{-1}
	Calcium-dependent cysteine-type endopeptidase activity	3	3.23	7.45^{-2}	A4I6E4, E9AHC1, A4I2N6		23	3190	6.50	9.6^{-1}	5.3^{-1}	5.3^{-1}
	Hydrolase activity	7	7.53	7.72^{-2}	A4HTD0, A4I7K4, A4IBE6, A4IBA6, A4I1Y3, A4I9R3, A4HWS5		152	3190	2.30	9.6^{-1}	5.3^{-1}	5.3^{-1}

Count: Number of gene names involved in a specific annotation term.
%: Genes belonging to an annotation term/total genes in the table.
p-value (probability value): Probability that a null hypothesis happens.
Genes: UniProt accession codes of the "count" belonging to an annotation term.
List total: Number of total genes detected by DAVID for a specific category.
Pop hits (population hits): Genes involved in a specific term from the whole *L. infantum* genome detected by DAVID.
Pop total (population total): The *L. infantum* genome background detected by DAVID in each category.
Fold enrichment: Percentage of genes within the total list annotated for a specific term divided by the percentage of genes annotated for the same term in the background (count/list total)/(pop hits/pop total).
Bonferroni: Bonferroni Šidák *p*-value (2).
Benjamini: Use of the linear step-up method of Benjamini and Hochberg to calculate the adjusted *p*-values (3).
FDR (false discovery rate): Expected proportion of false positives.

Supplementary Table S3. *L. infantum* proteins containing the peptides DNFIFGQ and AISVFFLEP or a part of them, with a minimum threshold of 5 continuous amino acids. Data obtained using the Basic Local Alignment Search Tool from the National Center for Biotechnology Information (https://blast.ncbi.nlm.nih.gov/Blast.cgi) and TriTrypDB (htpps://tritrypdb.org).

Peptide	Part of the peptide	Protein and gene name	Molecular function	Biological process	Cellular component
DNFIFGQ	DNFIFGQ	Beta tubulin (LINF_330015200)	GTPase activity, GTPase binding, structural constituent of cytoskeleton	Microtubule-based process	Microtubule
	FIFGQ	Oligosaccharyl transferase-like protein (LINF_350016300)	Oligosaccharyl transferase activity	Protein glycosylation	Nuclear envelope, ER, membrane
AISVFFLEP	AISVFFLEP	CPSF-like protein (LINF_320019100)	Nucleic acid binding, protein binding	N/A	Nucleus
	SVFFLE	Multi drug resistance protein-like (LINF_240020050)	ATP binding, ATPase-coupled transmembrane activity	Transmembrane transport	Integral component of membrane
	VFFLEP	Permease-like protein (LINF_230009700)	N/A	N/A	Membrane
	FFLEP	Conserved hypothetical protein (LINF_350051200)	Protein binding	N/A	N/A
	VFFLE	Neutral sphingomyelinase activation associated factor-like protein (LINF_230026400)	Protein binding	N/A	N/A
	VFFLE	BRE1 E3 ubiquitin ligase – putative (LINF_330012700)	N/A	Axoneme assembly	N/A
	VFFLE	Phosphate-repressible phosphate permease-like protein (LINF_030009800)	Inorganic phosphate transmembrane transporter activity	Phosphate ion transport	Membrane
	VFFLE	Conserved hypothetical protein (LINF_160008800)	N/A	N/A	N/A
	ISVFF	Conserved hypothetical protein (LINF_060007500)	N/A	N/A	Axoneme
	SVFFL	Dynein heavy chain – putative (LINF_340049400)	ATP binding, minus-end-directed microtubule motor activity	Microtubule-based movement, mitotic spindle organization, chromosome segregation, minus-end-directed vesicle transport along microtubule	Axoneme, dynein complex
	SVFFL	Hypothetical protein – conserved (LINF_300020000)	N/A	N/A	N/A
	SVFFL	Putative phosphatidylinositol 3-kinase 2 (LINF_140005100)	Kinase activity	Phosphatidylinositol phosphate biosynthetic process	N/A
	AISVF	HECT-domain (ubiquitin-transferase) – putative (LINF_340041000)	Ubiquitin-protein transferase activity	N/A	N/A
	AISVF	Enriched in surface-labeled proteome protein 11 – putative (LINF_340018700)	N/A	N/A	Ciliary pocket

N/A: not annotated data.

References

1. Varadi M, Anyango S, Deshpande M, Nair S, Natassia C, Yordanova G, Yuan D, Stroe O, Wood G, Laydon A, Žídek A, Green T, Tunyasuvunakool K, Petersen S, Jumper J, Clancy E, Green R, Vora A, Lutfi M, Figurnov M, Cowie A, Hobbs N, Kohli P, Kleywegt G, Birney E, Hassabis D, Velankar S 2022. AlphaFold Protein Structure Database: massively expanding the structural coverage of protein-sequence space with high-accuracy models. Nucleic Acids Res 50:D439-D444.
2. Šidák Z 1967. Rectangular confidence regions for the means of multivariate normal distributions. J Am Stat Assoc 62:626-633.
3. Benjamini Y, Hochberg Y 1995. Controlling the false discovery rate: a practical and powerful approach to multiple testing. J Roy Statist Soc Ser 57:289-300.
